# Cell Wall Carbohydrate Dynamics during the Differentiation of Infection Structures by the Apple Scab Fungus, Venturia inaequalis

**DOI:** 10.1128/spectrum.04219-22

**Published:** 2023-04-11

**Authors:** Mercedes Rocafort, Vaibhav Srivastava, Joanna K. Bowen, Sara M. Díaz-Moreno, Yanan Guo, Vincent Bulone, Kim M. Plummer, Paul W. Sutherland, Marilyn A. Anderson, Rosie E. Bradshaw, Carl H. Mesarich

**Affiliations:** a Laboratory of Molecular Plant Pathology, School of Agriculture and Environment, Massey University, Palmerston North, New Zealand; b Division of Glycoscience, Department of Chemistry, School of Engineering Sciences in Chemistry, Biotechnology and Health, Royal Institute of Technology (KTH), AlbaNova University Centre, Stockholm, Sweden; c The New Zealand Institute for Plant and Food Research Limited, Mount Albert Research Centre, Auckland, New Zealand; d Laboratory of Molecular Plant Pathology, School of Natural Sciences, Massey University, Palmerston North, New Zealand; e School of Food, Agriculture and Wine, The University of Adelaide, Waite Campus, Adelaide, South Australia, Australia; f Department of Animal, Plant and Soil Sciences, AgriBio, Centre for AgriBiosciences, La Trobe University, Bundoora, Melbourne, Victoria, Australia; g Department of Biochemistry and Genetics, La Trobe Institute for Molecular Science, La Trobe University, Bundoora, Melbourne, Victoria, Australia; h Bioprotection Aotearoa, Massey University, Palmerston North, New Zealand; Beijing Forestry University

**Keywords:** *Venturia inaequalis*, apple scab, cell wall, morphological differentiation, subcuticular infection structures

## Abstract

Scab, caused by the biotrophic fungal pathogen Venturia inaequalis, is the most economically important disease of apples. During infection, *V. inaequalis* colonizes the subcuticular host environment, where it develops specialized infection structures called runner hyphae and stromata. These structures are thought to be involved in nutrient acquisition and effector (virulence factor) delivery, but also give rise to conidia that further the infection cycle. Despite their importance, very little is known about how these structures are differentiated. Likewise, nothing is known about how these structures are protected from host defenses or recognition by the host immune system. To better understand these processes, we first performed a glycosidic linkage analysis of sporulating tubular hyphae from *V. inaequalis* developed in culture. This analysis revealed that the *V. inaequalis* cell wall is mostly composed of glucans (44%) and mannans (37%), whereas chitin represents a much smaller proportion (4%). Next, we used transcriptomics and confocal laser scanning microscopy to provide insights into the cell wall carbohydrate composition of runner hyphae and stromata. These analyses revealed that, during subcuticular host colonization, genes of *V. inaequalis* putatively associated with the biosynthesis of immunogenic carbohydrates, such as chitin and β-1,6-glucan, are downregulated relative to growth in culture, while on the surface of runner hyphae and stromata, chitin is deacetylated to the less-immunogenic carbohydrate chitosan. These changes are anticipated to enable the subcuticular differentiation of runner hyphae and stromata by *V. inaequalis*, as well as to protect these structures from host defenses and recognition by the host immune system.

**IMPORTANCE** Plant-pathogenic fungi are a major threat to food security. Among these are subcuticular pathogens, which often cause latent asymptomatic infections, making them difficult to control. A key feature of these pathogens is their ability to differentiate specialized subcuticular infection structures that, to date, remain largely understudied. This is typified by Venturia inaequalis, which causes scab, the most economically important disease of apples. In this study, we show that, during subcuticular host colonization, *V. inaequalis* downregulates genes associated with the biosynthesis of two immunogenic cell wall carbohydrates, chitin and β-1,6-glucan, and coats its subcuticular infection structures with a less-immunogenic carbohydrate, chitosan. These changes are anticipated to enable host colonization by *V. inaequalis* and provide a foundation for understanding subcuticular host colonization by other plant-pathogenic fungi. Such an understanding is important, as it may inform the development of novel control strategies against subcuticular plant-pathogenic fungi.

## INTRODUCTION

Scab, caused by the fungus Venturia inaequalis, is one of the most devastating diseases of apples worldwide (1, 2). Under favorable conditions, disease symptoms emerge as brown-green lesions on leaves, buds, and fruit, rendering the fruit unmarketable and reducing crop yield by up to 70% (3, 4). Scab is also the most expensive disease of apples to control, with up to 20 fungicide treatments required each year (4, 5). This intensive fungicide use has accelerated the development of fungicide resistance in *V. inaequalis* and has increased production costs for growers (6). While some disease-resistant apple cultivars have been developed, their use has been limited due to the rapid emergence of resistance-breaking strains of *V. inaequalis* in the field (7). Thus, there is an urgent need to develop durable control strategies against scab.

*V. inaequalis* is a biotrophic pathogen that colonizes the subcuticular (apoplastic) host environment located between the cuticle and underlying epidermal cells of apple tissues (1, 4). During colonization, *V. inaequalis* develops specialized subcuticular infection structures called runner hyphae and stromata (8–10). These structures are nonmelanized and are different from regular tubular hyphae developed on the host surface (9). Indeed, runner hyphae are wider and flatter than regular tubular hyphae and are often fused along their length to form “hyphal superhighways” (9), while stromata are multilayered pseudoparenchymatous structures that are the result of a switch from a polar tip extension to nonpolar lateral division (9). In terms of functionality, stromata give rise to asexual conidia that further the infection cycle but are also thought to be involved in nutrient acquisition and effector (virulence factor) delivery. Runner hyphae, on the other hand, enable the fungus to radiate out from the initial site of host penetration, acting as a base from which additional stromata can be differentiated (9).

Notably, subcuticular infection structures are also produced by other crop-infecting members of the *Venturia* genus (11–14), as well as several other species of plant-pathogenic fungi. The latter include Diplocarpon rosae, which causes black spot disease of roses (15), and Rhynchosporium secalis, which causes barley and rye scald (16, 17). Despite these observations, very little is known about how these structures are differentiated or how the fungal cell wall is remodeled during this process. Strikingly, *V. inaequalis* can develop infection-like structures inside cellophane membranes (CMs) that are reminiscent of those formed *in planta* (9). This contrasts with growth on the CM surface, where the fungus develops tubular hyphae like those formed on the surface of apple tissues (9). This finding suggests that CMs can be used as an in-culture model for studying the differentiation of subcuticular infection structures and the dynamics of cell wall remodeling.

The fungal cell wall is an external barrier that plays an essential role in fungal growth and morphogenesis (18, 19). For plant-pathogenic fungi, the cell wall also has an important role in protection, as it is the first structure to encounter the hostile apoplastic environment of the host (20). Despite this importance, its composition and biosynthesis are still poorly understood, especially for non-model filamentous pathogens (19). While the structures and compositions of the fungal cell wall differ between species, the wall is typically comprised of a polysaccharide and protein matrix, with glucans, chitin, and mannans the main components (18). Crucially, some of these carbohydrates, such as chitin and β-glucan, are strong elicitors of the plant immune system, with defense responses initiated upon their recognition as microbe-associated molecular patterns (MAMPs) by cell-surface-localized plant immune receptors (21, 22).

Chitin, a linear polymer of β-1,4-linked *N*-acetylglucosaminyl residues, is synthesized by membrane-bound glycosyltransferase (GT) family 2 enzymes called chitin synthases (CHSs) (18, 23). In terms of glucans, the majority are of the β-1,3-linked type, which in some instances is cross-linked with chitin to form the core structure of the fungal cell wall. Additionally, different proportions of branched β-1,6-glucan can be found in some fungi, usually extending to the cell wall surface, where it forms connections to mannoproteins (19, 24, 25). β-1,3-Glucan is synthesized by a membrane-bound enzyme from GT family 48 (GT48) (25, 26). The enzymes required for β-1,6-glucan biosynthesis have not been described in any filamentous fungal species (27). However, multiple enzymes associated with β-1,6-glucan biosynthesis and β-1,3-glucan modification have been described in the yeast Saccharomyces cerevisiae (18, 27, 28).

Given the immunogenic nature of some cell wall carbohydrates, plant-associated fungi must modify their cell wall during host colonization to avoid detection (29, 30). Likewise, as the apoplast is rich in plant-derived glucanases and chitinases (29), fungi must also actively prevent the hydrolytic release of chitin and β-glucan oligomers from their cell walls (31). One proposed strategy used by fungi is to deacetylate chitin to chitosan (32–35), which is a poor elicitor of plant defenses (36–38) and a weak substrate of plant chitinases (39, 40). Another strategy is to accumulate α-1,3-glucan on the cell surface, which shields it from the action of plant hydrolases and, in doing so, prevents the release of carbohydrate-based MAMPs (41–44).

In line with the importance of the fungal cell wall, its structure and the enzymes required for its biosynthesis are common targets for antifungal compounds (24, 45). As such, knowledge of fungal cell wall carbohydrate composition is important for the development of novel fungicides and, therefore, control strategies. To date, a detailed analysis of the cell wall carbohydrate composition in a *Venturia* species has not been published, with only one study in 1965 reporting that the cell wall of *V. inaequalis* grown in culture is made up of 28% hemicellulose, 13% β(γ)-cellulose, 20% α-cellulose, and 7% chitin (46). As cellulose is generally accepted to be absent from fungal cell walls (47), a more thorough investigation of the cell wall carbohydrate composition in *V. inaequalis* using state-of-the-art techniques is now needed. Here, using a glycosidic linkage analysis, with support from gene expression and proteomic data, we report the cell wall carbohydrate composition of sporulating tubular hyphae from *V. inaequalis* developed on the surface of CMs. Then, using confocal laser scanning microscopy (CLSM), again in conjunction with gene expression data, we provide insights into the cell wall carbohydrate composition of subcuticular infection structures developed by *V. inaequalis in planta* and compare these to the infection-like structures developed in CMs.

## RESULTS

### The major cell wall polysaccharides of sporulating tubular hyphae formed by *V. inaequalis* in culture are glucans and mannans.

**FIG 1:**
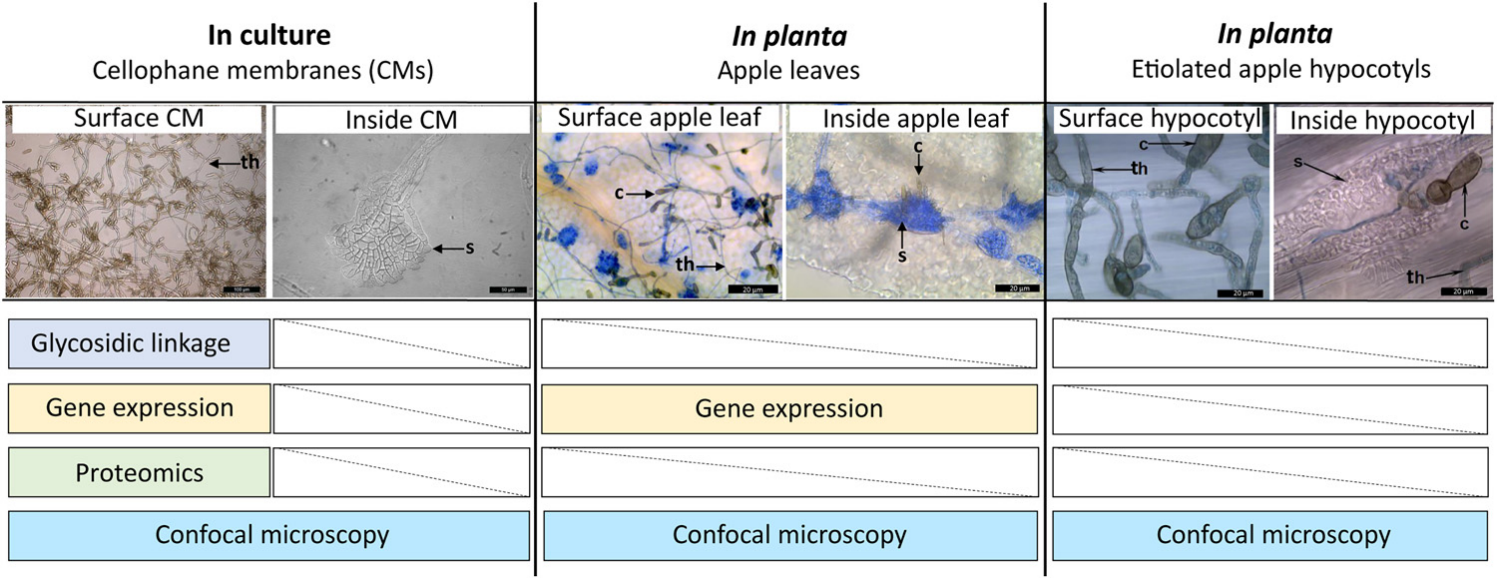
Summary of *Venturia inaequalis* samples used in this study. Tubular hyphae growing on the surface of cellophane membranes (CMs) overlaying potato dextrose agar were used for the glycosidic linkage analysis, proteomic analysis, and gene expression (RNA-seq) analysis, as well as confocal laser scanning microscopy (CLSM). Infection-like structures formed inside CMs were used for CLSM. Infected apple leaves were used for the gene expression analysis and CLSM. Infected etiolated apple hypocotyls (a model *in planta* infection system) were used for CLSM. All scale bars are 20 μm. c, conidium; s, stroma; th, tubular hypha.

**FIG 2:**
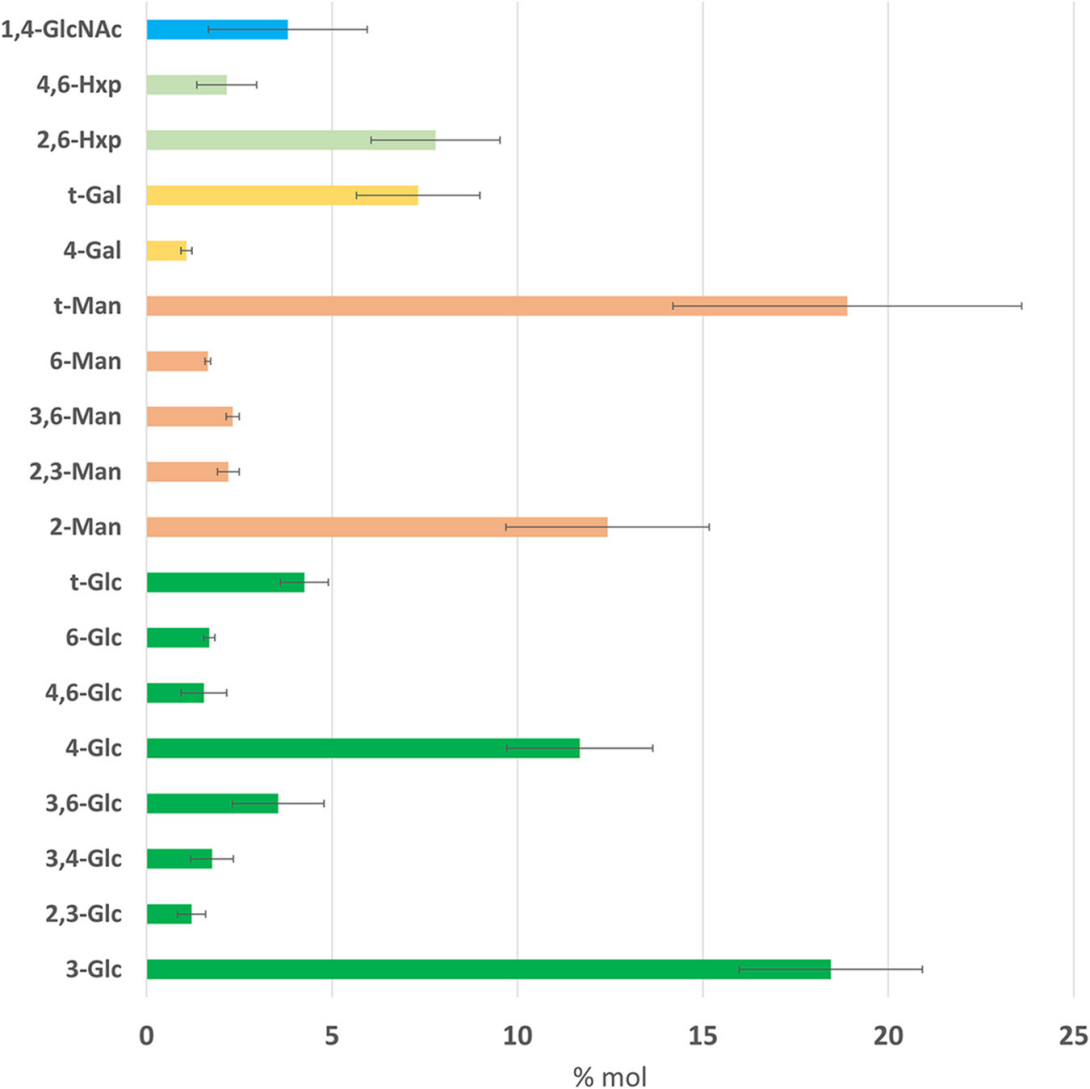
Glycosidic linkage analysis (moles percent [% mol]) of the cell wall carbohydrate fraction from sporulating tubular hyphae of Venturia inaequalis developed on the surface of cellophane membranes (CMs) overlaying potato dextrose agar at 5 days postinoculation. Man, mannose; Glc, glucose; Gal, galactose; Hxp, hexopyranose; GlcNAc, *N*-acetylglucosamine. Error bars represent standard deviation across three technical replicates.

To investigate the carbohydrate composition of the *V. inaequalis* cell wall during growth in culture, a glycosidic linkage analysis was performed on cell walls harvested from sporulating tubular hyphae developed on cellophane membranes (CMs) overlaying potato dextrose agar (PDA) (Fig. 1). (From this point forward, we refer to growth on CMs as in-culture growth.) The fungal material was extensively washed to avoid contamination from the underlying PDA. The glycosidic linkage analysis revealed that most polysaccharides present in the *V. inaequalis* cell wall were composed of glucosyl (Glc) (~44%) and mannosyl (Man) (~37%) residues, followed by unidentified hexopyranosyl (Hxp) (~10%), galactosyl (Gal) (~8%), and *N*-acetylglucosaminosyl (GlcNAc) (~4%) residues (Fig. 2). This analysis also revealed that the most dominant Glc linkage was 1,3-Glc (41.7%), followed by 1,4-Glc (26%), terminal Glc (t-Glc) (9.63%), 1,3,6-Glc (8.03%), 1,6-Glc (3.8%), and 1,4,6-Glc (3.5%) (Fig. 2). The entire GlcNAc fraction consisted of 1,4-GlcNAc residues, while the most dominant Man linkage was t-Man (50.4%), followed by 1,2-Man (33.1%). Finally, the Hxp fraction consisted of only two linkages, 2,6-Hxp (78.3%) and 4,6-Hxp (21.7%), while the Gal fraction was mostly t-Gal (87.2%) and 1,4-Gal (12.8%) (Fig. 2).

### Identification and expression of genes putatively associated with cell wall polysaccharide biosynthesis in sporulating tubular hyphae of *V. inaequalis*.

**FIG 3:**
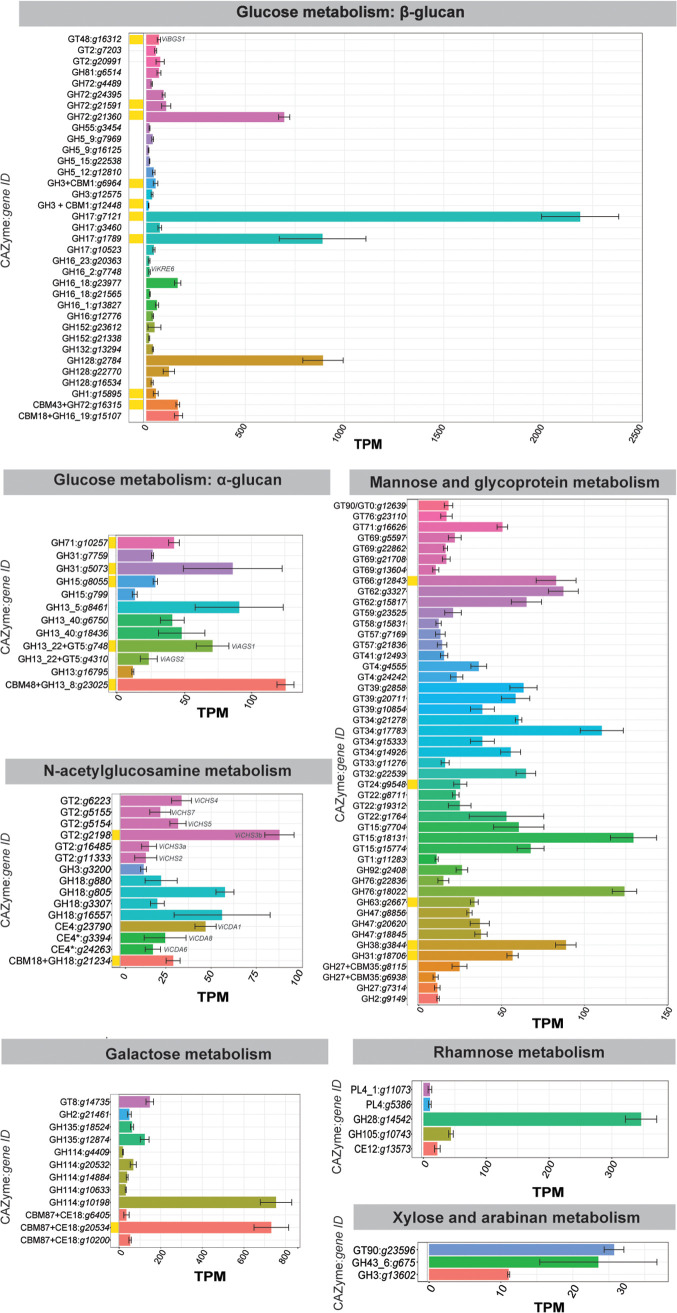
Expression of carbohydrate-active enzyme (CAZyme)-encoding genes from Venturia inaequalis putatively associated with cell wall biosynthesis during growth as sporulating tubular hyphae on the surface of cellophane membranes overlying potato dextrose agar at 7 days postinoculation. Gene expression data are transcripts per million (TPM), averaged from four biological replicates, with error bars representing standard deviation. Only genes with TPM values of >10 are shown and are grouped into families by color. Enzymes labeled with an asterisk were identified by Protein family (Pfam) search. Yellow blocks indicate proteins that have proteomic support by mass spectrometry. AGS, α-1,3-glucan synthase; BGS, β-1,3-glucan synthase; CBM, carbohydrate-binding module; CDA, chitin deacetylase; CE, carbohydrate esterase; CHS, chitin synthase; GH, glycoside hydrolase; GT, glycosyltransferase; PL, polysaccharide lyase.

To determine which cell wall carbohydrate biosynthetic genes are expressed during growth of *V. inaequalis* on the surface of CMs, we used a combination of bioinformatic, proteomic, and transcriptomic approaches (Fig. 1). As a starting point, the most recently predicted gene catalogue for *V. inaequalis* (48) was inspected to identify genes putatively associated with cell wall biosynthesis (see Table S1 and Supplemental File 1 in the supplemental material). Based on this analysis, 231 genes were identified (Supplemental File 1). Next, the expression of these genes was investigated using preexisting RNA-seq data from sporulating tubular hyphae of *V. inaequalis* grown on CMs at 7 days postinoculation (dpi) (48). This analysis revealed that, of the 231 predicted cell wall biogenesis genes, 135 were expressed with a value of >10 transcripts per million (TPM) (Fig. 3). Finally, a proteomic analysis of total protein from sporulating tubular hyphae of *V. inaequalis* grown on CMs at 5 dpi was performed. Based on this analysis, 24 of the 231 putative cell wall biosynthetic genes were found to encode proteins with proteomic support (Supplemental Files 3 and 4), confirming that they were indeed produced. Of these, 17 had a TPM expression value of >10 (Fig. 3).

**FIG 4:**
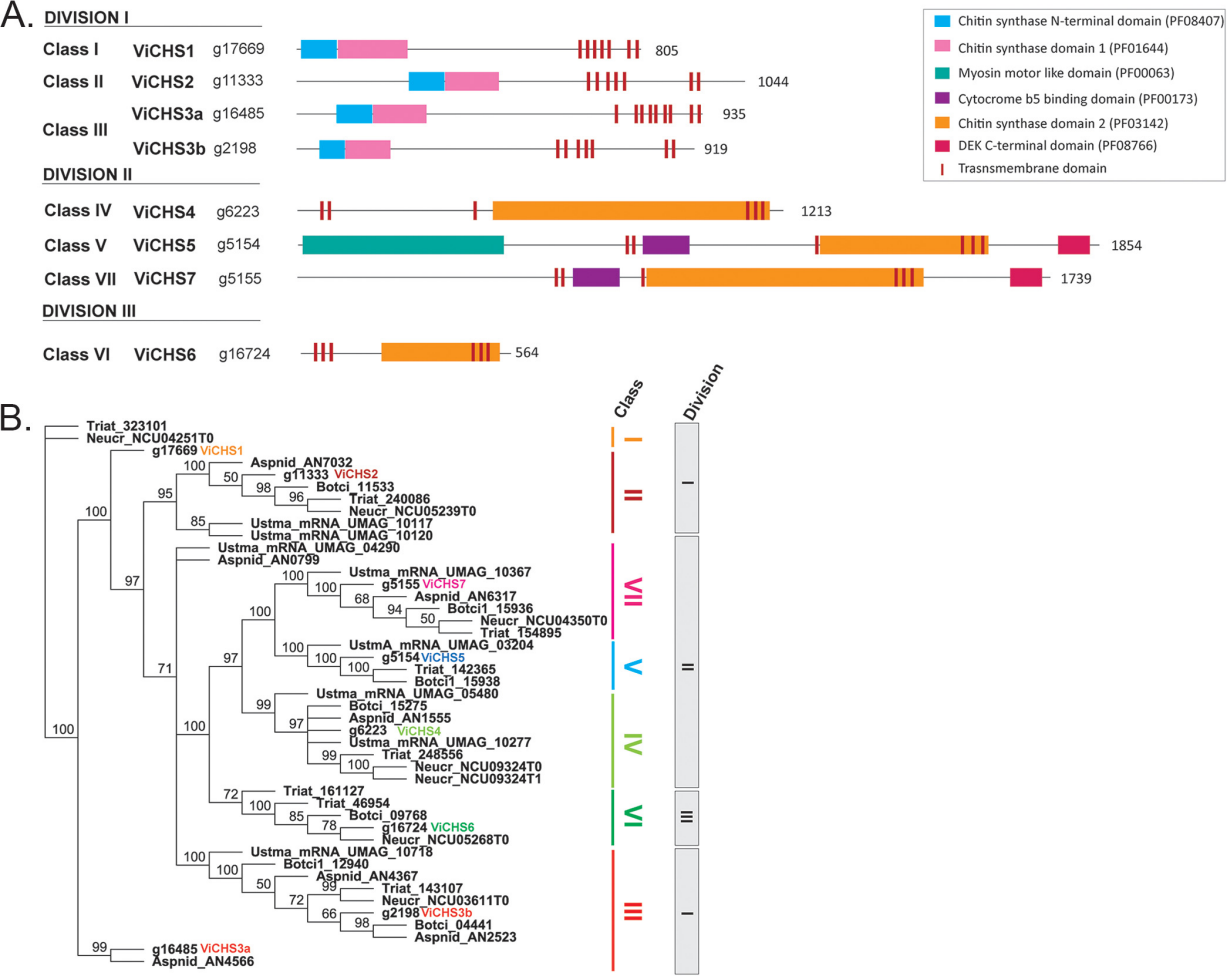
Chitin synthase (CHS) proteins of Venturia inaequalis. (A) Predicted classification and domain organization of CHS proteins; (B) phylogenetic classification of the eight predicted CHSs. CHSs from Aspergillus nidulans (Aspnid), Neurospora crassa (Neucr), Botrytis cinerea (Botci), and Ustilago maydis (Ustma) were included for reference. *V. inaequalis* CHS proteins are highlighted in boldface and colored letters. Phylogenetic tree was generated from a MUSCLE alignment and end-joining method using Geneious v.9.0.5, with node values indicating consensus support (percentage) from 100 replicates.

Among the glycosyltransferases (GTs) identified, 13 were putative family 2 enzymes and, of these, eight were annotated as CHSs. These were named ViCHS1 to -7 according to the previously established CHS classification scheme (49), based on both their CHS domain (Fig. 4A) and phylogenetic distribution (Fig. 4B). *V. inaequalis* had at least one representative from each CHS class (classes I to VI) and two CHSs from class III (Fig. 4A). Four CHSs were from division I, having a simple amino-terminal (N-terminal) CHS domain 1 plus CHS domain 1 structure (Fig. 4A). All division I CHSs, except ViCHS1, were encoded by genes that had a TPM expression value of >10, with *ViCHS3b* being the most highly expressed and the only *CHS* gene with proteomic support (Fig. 3). Three CHSs were from division II and, of these, ViCHS4 only had a single CHS domain 2 module (Fig. 4A). In contrast, ViCHS5 and ViCHS7 both had a cytochrome *b*_5_-binding domain and a Dek domain at their carboxyl terminus (C terminus), while ViCHS5 also had an N-terminal myosin motor-like domain (Fig. 4A). Finally, ViCHS6 was from division III and had a single C-terminal CHS domain 2 module (Fig. 4A). The gene encoding this CHS, however, was not highly expressed in culture (TPM value of <10). All CHSs contained both a QxxRW motif required for catalytic activity as well as QxxEY and EDRxL domains of unknown function (Fig. S1) (23).

Multiple enzymes putatively associated with chitin modification were encoded by genes expressed during growth of *V. inaequalis* in culture, including five glycoside hydrolase (GH) family 18 chitinases and one β-*N*-acetyl hexosaminidase of GH family 3 (Fig. 3; Supplemental File 1). However, only one chitinase had proteomic support (Fig. 3). In total, eight putative chitin deacetylase (*CDA*) genes were identified (Supplemental File 1). Of those (Fig. S2A), a sequence alignment revealed that only the encoded proteins ViCDA1 and ViCDA7 possessed all previously described conserved residues for catalytic activity and metal binding and were likely functional (50, 51) (Fig. S2B). ViCDA4 had all metal-binding and catalytic site residues, except one, as the second conserved aspartic acid for catalytic activity was substituted by a similar negatively charged amino acid, glutamic acid. Therefore, it is likely that ViCDA4 is functional. ViCDA5 and ViCDA6 had all catalytic site residues, except the last histidine, and an amino substitution in the first metal-binding position (Fig. S2B). As such, these two proteins might not be functional. ViCDA3 had two amino acid substitutions at the first and third metal-binding positions, which are characteristic of allantoinases; enzymes that hydrolyze allantoin, a nitrogen-rich organic compound (52) (Fig. S2B). Therefore, ViCDA3 might function as an allantoinase instead of a CDA. Finally, due to a C-terminal truncation, ViCDA8 was missing the last two catalytic site residues and, therefore, is likely to be nonfunctional (Fig. S2B). None of the CDAs had a predicted transmembrane domain or glycosylphosphatidylinositol (GPI) anchor, while only ViCDA1 and ViCDA4 had a predicted N-terminal signal peptide for secretion (Fig. S2A). *ViCDA1* was the only potentially active CDA-encoding gene with a high level of expression during growth of *V. inaequalis* in culture; however, this enzyme did not have proteomic support (Fig. 3; Fig. S2C).

Only one gene encoding a putative β-1,3-glucan synthase of GT family 48, *ViBGS1*, was identified in the *V. inaequalis* genome (Supplemental File 1). This gene was expressed in culture, and the resulting enzyme was detected by the proteomic analysis (Fig. 3). Additionally, many genes encoding β-1,3-glucan-modifying enzymes were identified, such as members of the GH17 and GH72 families (Supplemental File 1), which are anticipated to assist in either the elongation or branching of β-1,3-glucans (28, 53). Of these, four *GH17* genes and four *GH72* genes were expressed during growth of *V. inaequalis* in culture and half of these had proteomic support (Fig. 3). This included the *GH17* gene *g7121*, which was the most highly expressed carbohydrate-active enzyme (CAZyme)-encoding gene.

A further 13 genes encoding putative GH family 16 enzymes were also identified (Supplemental File 1), with four of these found to be expressed during growth in culture (Fig. 3). Three of these possibly encode chitin transglycosylases required to cross-link chitin with glucan (54), while the fourth encodes a KRE6-like enzyme (ViKRE6) that is possibly associated with β-1,6-glucan biosynthesis (55, 56). None of the four GH16 enzymes was identified in the proteomic analysis.

The genome of *V. inaequalis* also carried two genes encoding putative α-1,3-glucan synthases, named *ViAGS1* and *ViAGS2* (Supplemental File 1). Both were expressed in culture; however, only the *ViAGS1* gene had proteomic support (Fig. 3). Finally, the *V. inaequalis* genome possessed 71 genes involved in the biosynthesis of mannan (Supplemental File 1). Of these, six encoded mannan polymerases and 25 encoded mannosyltransferases expressed with a TPM value of >10 in culture. However, none of these enzymes had proteomic support (Fig. 3).

### Genes putatively associated with the biogenesis of carbohydrate-based MAMPs from *V. inaequalis* are downregulated during host colonization.

**FIG 5:**
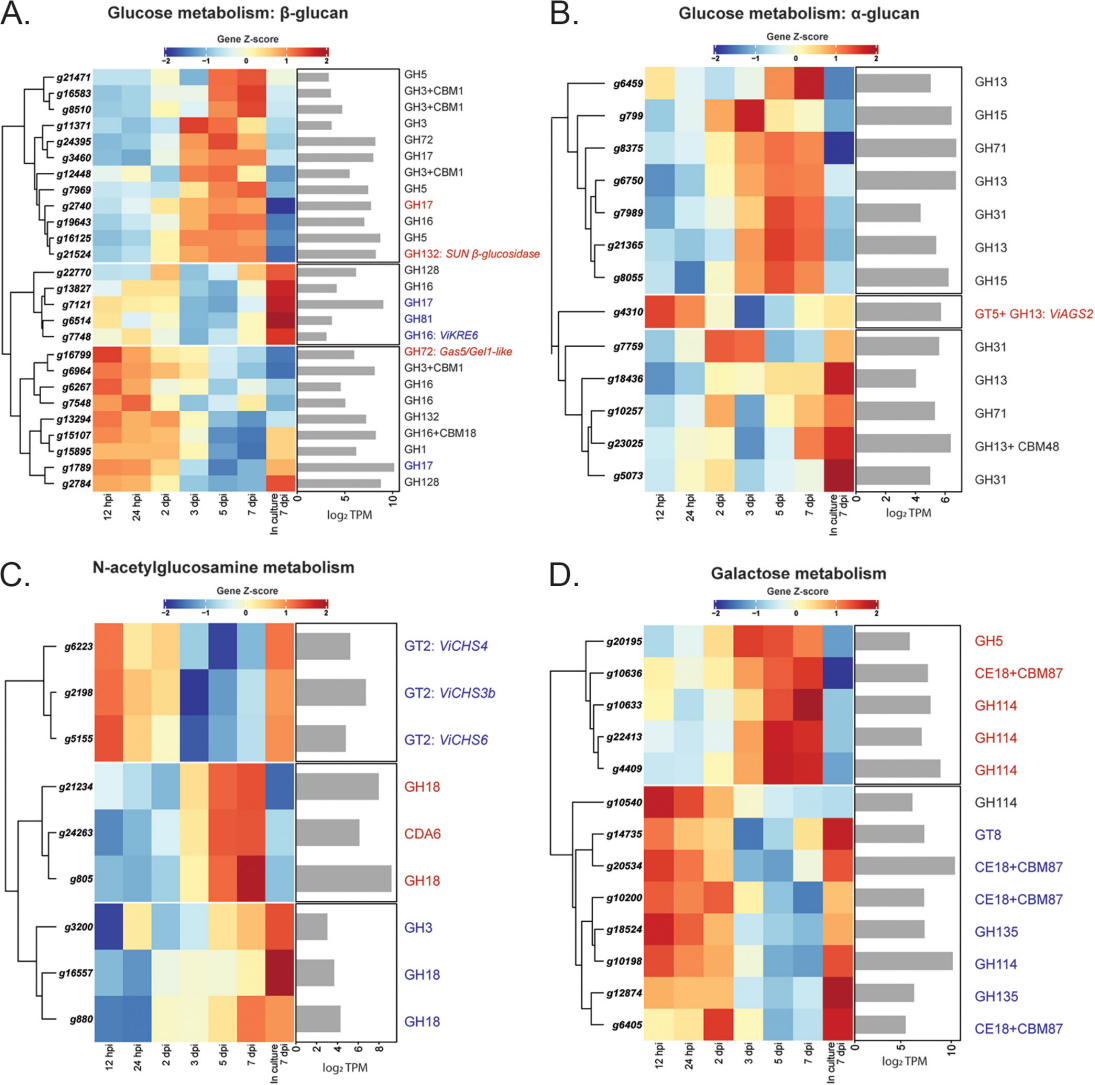
Heat maps showing the expression profiles of genes from Venturia inaequalis that are both putatively associated with cell wall biosynthesis and differentially expressed during host colonization relative to growth in culture. Only differentially expressed genes putatively associated with (A) glucose (β-glucan), (B) glucose (α-glucan), (C) *N*-acetylglucosamine, and (D) galactose metabolism at one or more *in planta* time points compared to growth on cellophane membranes overlying potato dextrose agar and with a minimum expression of 10 transcripts per million (TPM) are shown. Heat maps are rlog-normalized counts across all samples (Z-score), averaged from four biological replicates. Bar plots depict the maximum log_2_ TPM count value across all *in planta* time points. hpi, hours postinoculation; dpi, days postinoculation; CBM, carbohydrate-binding module; CDA, chitin deacetylase; CE, carbohydrate esterase; CHS, chitin synthase; GH, glycoside hydrolase; GT, glycosyltransferase. Blue highlights genes downregulated and red highlights genes upregulated during host colonization relative to growth in culture.

A glycosidic linkage analysis could not be performed to determine the carbohydrate composition of subcuticular infection structures produced by *V. inaequalis* during infection of apple tissue. This was due to the paucity of fungal material generated during subcuticular growth. Thus, to make inferences about how the *V. inaequalis* cell wall carbohydrate composition changes during host infection, relative to growth in culture, the expression of the 231 putative cell wall biosynthesis genes identified above was investigated using preexisting *in planta* transcriptomic data (48) (Fig. 1). These data were collected from apple leaves infected with *V. inaequalis* at six time points: 12 and 24 h postinoculation (hpi), as well as 2, 3, 5 and 7 dpi (48). Using these data, a total of 68 genes putatively associated with fungal cell wall biosynthesis were found to be upregulated, and 43 downregulated, at one or more *in planta* time points compared to growth in culture (Supplemental File 2). Interestingly, most differentially expressed genes were associated with β-glucan metabolism (Fig. 5A; Fig. S3). More specifically, during early infection (12 to 24 hpi), the *Gas5/Gel1-like* gene, which encodes a GH72 1,3-β-glucanosyltransferase with sequence similarity to Gas5 enzymes from yeast and Gel1 from Aspergillus spp. (57, 58), was upregulated. Later, during mid to late infection (5 and 7 dpi), several β-glucosidase-encoding genes were upregulated. Only a few genes associated with β-glucan metabolism were downregulated during host colonization, such as two genes encoding GH17 proteins and the KRE6-like enzyme (Fig. 5A; Fig. S3). Regarding α-1,3-glucan metabolism, *ViAGS2* was upregulated during early (12 and 24 hpi) host infection (Fig. 5B). Importantly, as a validation of the RNA-seq data, downregulation of *ViKRE6* and upregulation of *ViAGS2* during early infection were confirmed by real-time quantitative PCR (RT-qPCR) (Fig. S4).

Genes involved in chitin metabolism were, in general, downregulated during host colonization (Fig. 5C; Fig. S5). During early infection, two genes encoding putative GH18 chitinases and one gene encoding a GH3 enzyme of unknown function, were downregulated. Later, during mid to late infection, the *CHS* genes *ViCHS3b*, *ViCHS4*, and *ViCHS7* were downregulated. Interestingly, from 3 dpi, two chitinase-encoding genes, as well as the gene encoding the putatively inactive ViCDA6, were upregulated (Fig. 5C; Fig. S5). Additionally, *ViCDA1*, which encodes the putatively secreted and active CDA, was constitutively expressed both in culture and *in planta* (Fig. S2C).

Several genes associated with galactose metabolism were downregulated during mid to late infection. However, others were upregulated over this infection stage, especially three genes encoding GH114 proteins (Fig. 5D). Finally, most genes associated with mannose and glycoprotein metabolism were upregulated, especially those genes encoding putative mannosidases (Supplemental File 2).

### Chitosan is present on the surface of subcuticular infection structures formed by *V. inaequalis in planta*, while chitin is restricted to septa.

To further investigate the cell wall carbohydrate composition of the cellular morphotypes formed by *V. inaequalis*, we used CLSM in conjunction with different carbohydrate-specific probes and antibodies to label chitin, chitosan, β-1,3-glucan, and α-1,3-glucan. CLSM was performed on *V. inaequalis* growing in culture on the surface and inside CMs, as well as during host colonization on the surface and inside living host tissue (Fig. 1). For host colonization, two approaches were taken, with the first involving detached apple leaves and the second involving detached etiolated apple hypocotyls (Fig. 1). Here, etiolated apple hypocotyls were included as they have previously been shown to be a good system for visualizing infection by *V. inaequalis* due to their reduced tissue thickness, as well as their lower levels of chlorophyll, other pigments, and phenolic compounds, compared to apple leaves (59). However, it is important to note that hypocotyl infection does not occur in orchards, as these apples are cultivated from clonal bud wood, not seed. Hypocotyl infection may occur, though, in natural apple forests.

**FIG 6:**
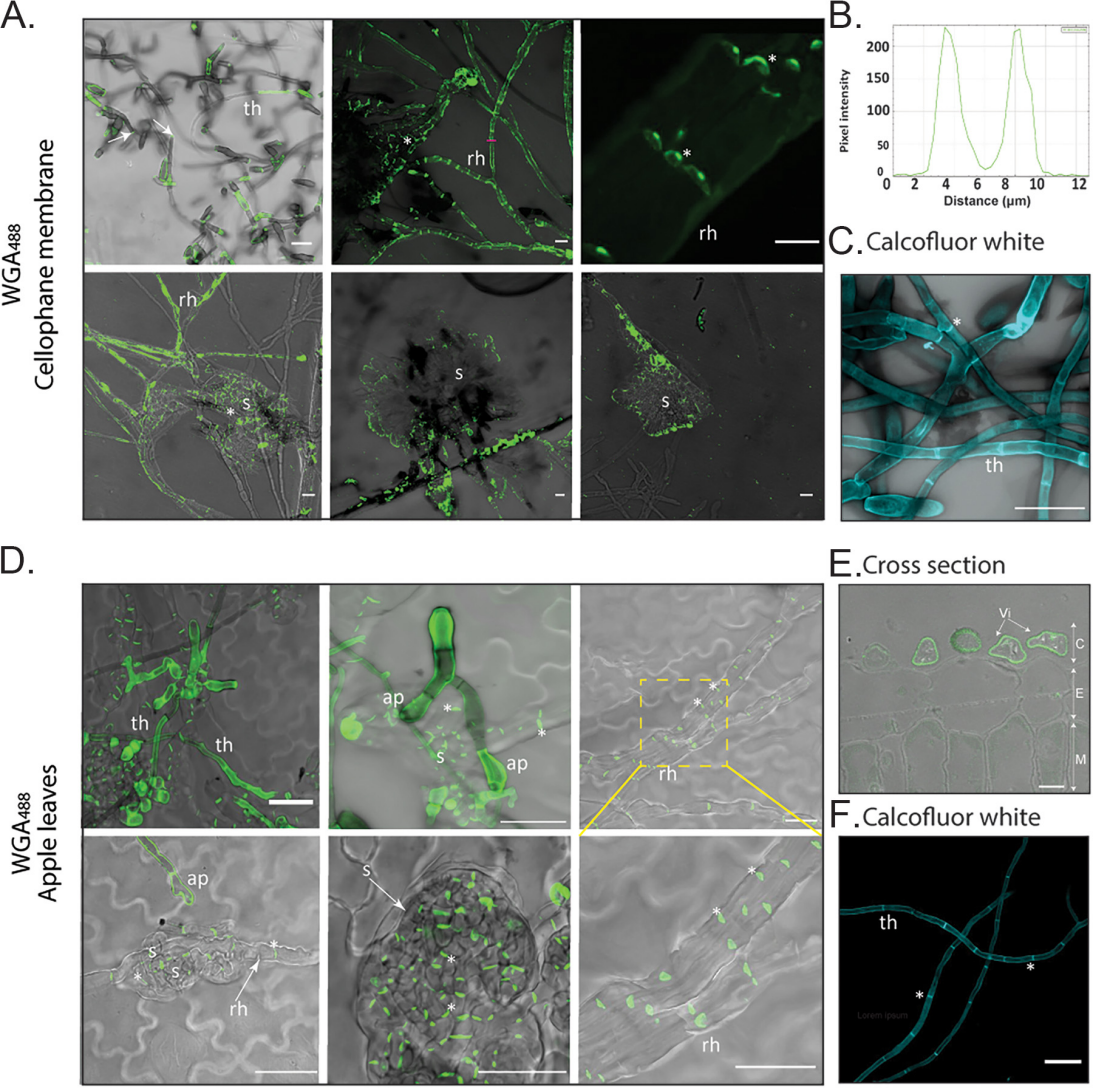
Labeling of chitin on Venturia inaequalis cellular morphotypes developed in culture and *in planta*. (A) Cellular morphotypes developed in culture in and on cellophane membranes (CMs) overlaying potato dextrose agar labeled with wheat germ agglutinin-Alexa Fluor 488 (WGA^488^) (green pseudocolor). Scale bars are 20 μm. (B) Plot profile of pixel intensity along a line (magenta) in panel A (top middle image) made with ImageJ 1.x. (C) Tubular hyphae developed on a CM labeled with calcofluor white (cyan pseudocolour) after permeabilization with NaOH. Scale bar is 20 μm. Dashed yellow squares indicates an enlarged area. (D) Cellular morphotypes developed in and on apple leaves labeled with WGA^488^ (green pseudocolor). (E) Chitin labeling of a cross section from an apple leaf infected with *V. inaequalis* using WGA^488^ (green pseudocolour). C, apple cuticle; E, apple epidermal cells; M, apple mesophyll cells; Vi, *V. inaequalis*. Scale bar, 5 μm. (F) Tubular hyphae developed on the surface of an apple leaf labeled with calcofluor white (cyan pseudocolour) after permeabilization with NaOH. Scale bar is 20 μm. ap, appressorium; rh, runner hyphae; s, stroma; th, tubular hypha. Arrows indicate hyphal breakage, and asterisks indicate septa.

To monitor the distribution of chitin on the surface of the fungal cell wall, all samples were labeled with wheat germ agglutinin (WGA) conjugated to the fluorophore Alexa Fluor 488 (AF488) (WGA^488^) (Fig. 6; Fig. S6). Tubular hyphae formed on the surface of CMs (Fig. 6A), infected apple leaves (Fig. 6D and F), and etiolated hypocotyls (Fig. S6) presented limited amounts of surface-exposed chitin, with labeling mainly restricted to hyphal breakage points and, sometimes, septa. The maximum fluorescence intensity occurred at the cell periphery, indicating that the labeling signal was derived from the cell wall (Fig. 6B). To label the total chitin present in the cell wall, fungal material was first permeabilized with NaOH and then labeled with the chitin stain calcofluor white. By doing so, faint labeling was observed at the periphery of tubular hyphae, with stronger labeling found at septa (Fig. 6C). Labeling was also observed at the periphery of *V. inaequalis* cells in cross sections of infected apple leaves using WGA^488^ (Fig. 6E). On the infection-like structures developed inside CMs, chitin labeling was observed around the periphery of runner hyphae, as well as at the septa of runner hyphae and stromata (Fig. 6A). In contrast, following host penetration, chitin was exclusively restricted to septa (Fig. 6D; Fig. S6), as has previously been shown for other plant-associated fungi (33, 34).

Next, as *V. inaequalis* has eight putative *CDA* genes (Fig. 5) and, of these, *ViCDA6* is upregulated during infection, while *ViCDA1* is constitutively expressed in culture and *in planta*, it was hypothesized that *V. inaequalis* deacetylates chitin to chitosan during host colonization. Therefore, the tubular hyphae and subcuticular infection structures of *V. inaequalis* developed *in planta*, as well as the tubular hyphae and infection-like structures of this fungus formed in culture, were probed using the oligogalacturonate (OGA) probe conjugated with AF488 (OGA^488^) (60).

**FIG 7:**
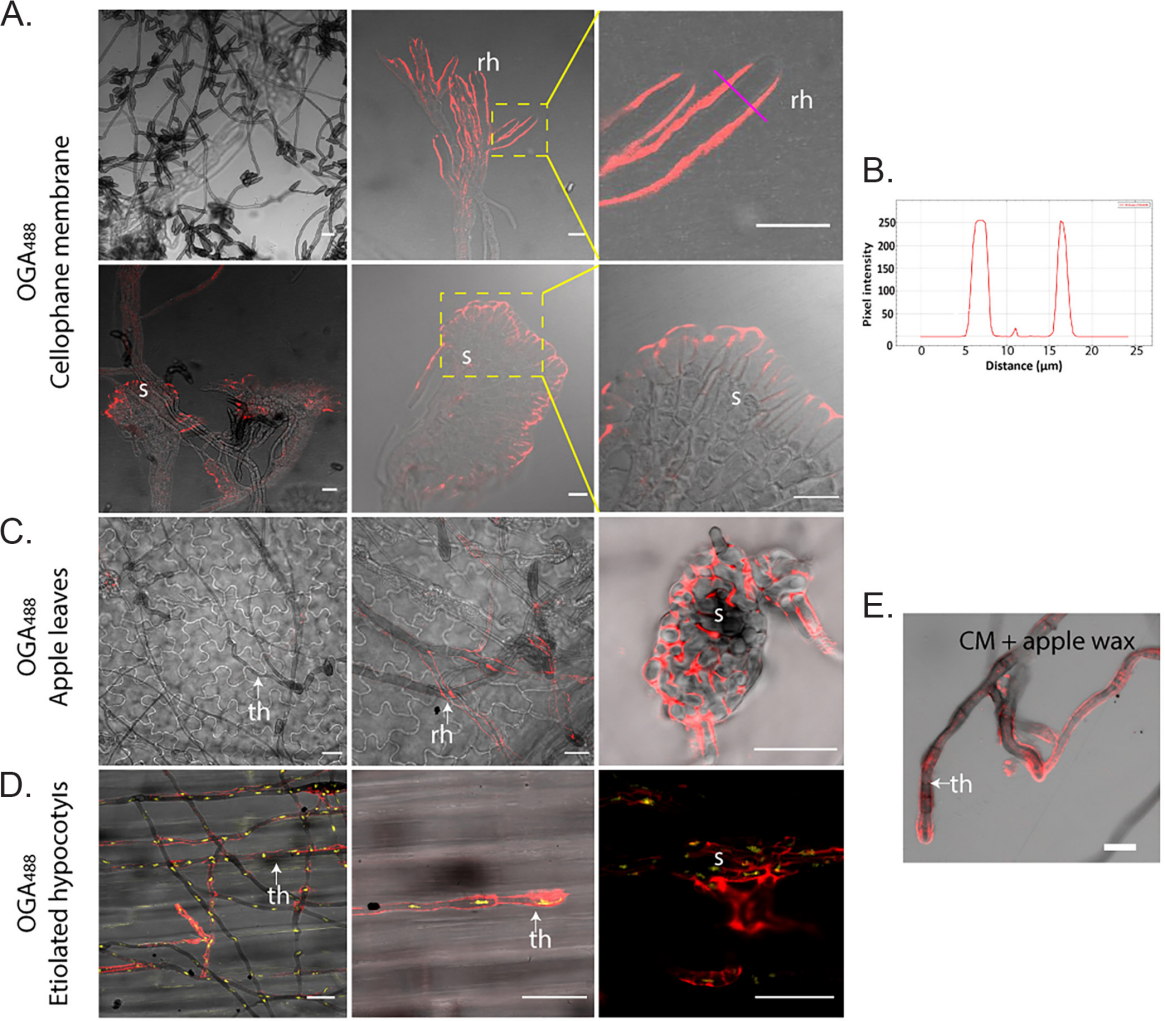
Label-accessible chitosan on the surface of Venturia inaequalis cellular morphotypes developed in culture and *in planta*. The fluorophore-labeled oligosaccharide OGA^488^ was used to visualize chitosan (red pseudocolour). (A) *V. inaequalis* cellular morphotypes developed in culture in and on cellophane membranes (CMs) overlaying potato dextrose agar. (B) Plot profile of pixel intensity along a line (magenta) in panel A (top right-hand image) made with ImageJ 1.x. (C) Cellular morphotypes developed in and on apple leaves. (D) Cellular morphotypes developed in and on etiolated hypocotyls, with fungal nuclei labeled with propidium iodide (PI) (yellow pseudocolor). (E) Surface hyphae developed on a CM covered with apple wax. All scale bars are 20 μm. ap, appressorium; rh, runner hyphae; th, tubular hypha; s, stroma. Dashed yellow squares indicate enlarged areas.

Chitosan labeling was not detected on tubular hyphae developed on the surface of CMs or apple leaves (Fig. 7A and C). In contrast, tubular hyphae developed on etiolated hypocotyls had surface-exposed chitosan (Fig. 7D), highlighting a key difference among these host infection systems. The reason behind chitosan induction on the surface of etiolated hypocotyls is not clear, but we hypothesize that *V. inaequalis* may grow more intimately with the cutin layer and, as part of this, there might be some plant-derived trigger (e.g., cutin monomers) inducing chitosan production. With this in mind, we tested if chitosan production could be induced in culture. For this purpose, wax was extracted from apple fruit and added to the surface of CMs before inoculation with *V. inaequalis* conidia. Remarkably, following germination, apple wax triggered chitosan production on tubular hyphae developed in culture (Fig. 7E). The maximum fluorescence intensity from chitosan labeling occurred at the cell periphery, indicating that the labeling signal was derived from the cell wall (Fig. 7B). Regarding the subcuticular infection structures developed *in planta*, as well as the infection-like structures developed in CMs, chitosan labeling was also observed at the periphery (Fig. 7C and D). Unfortunately, despite several attempts, we were unable to detect chitosan in the cell walls of *V. inaequalis* using cross sections of infected apple leaves in conjunction with OGA^488^.

**FIG 8:**
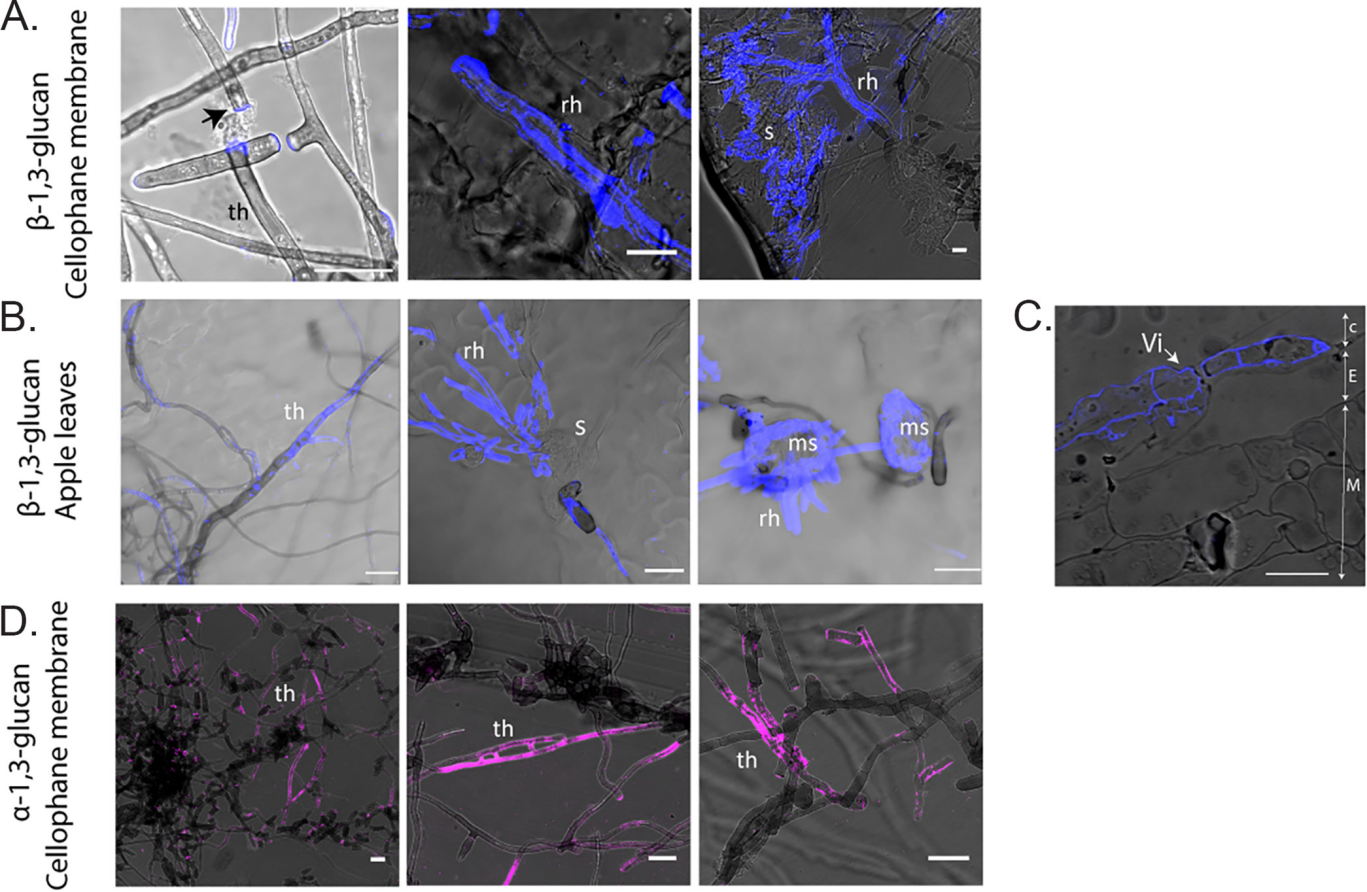
Label-accessible β-1,3-glucan and α-1,3-glucan of *Venturia inaequalis* cellular morphotypes developed in culture and *in planta*. The monoclonal anti β-1,3-glucan primary antibody and CF^488^ secondary antibody were used to label β-1,3-glucan (blue pseudocolor), while the monoclonal MOPC-104E primary antibody and CF^488^ secondary antibody were used to label α-1,3-glucan (pink pseudocolour). (A) β-1,3-Glucan labeling of *V. inaequalis* cellular morphotypes developed in culture in and on cellophane membranes (CMs). Scale bars are 20 μm. (B) β-1,3-Glucan labeling of *V. inaequalis* cellular morphotypes developed in and on apple leaves. Scale bars are 20 μm. (C) β-1,3-Glucan labeling of a cross section from an apple leaf infected with *V. inaequalis*. C, apple cuticle; E, apple epidermal cells; M, apple mesophyll cells; Vi, *V. inaequalis*. Scale bars are 10 μm. (D) α-1,3-Glucan labeling of *V. inaequalis* cellular morphotypes developed in culture in and on CMs. Scale bars are: 20 μm. ms, mature stroma; rh, runner hyphae; s, stroma; th, tubular hyphae. Black arrow indicates hyphal breakage point.

Next, we investigated the distribution of β-1,3-glucan during growth of *V. inaequalis* in culture and *in planta*, as β-1,3-glucan is known to be an elicitor of plant defenses (61). For this purpose, β-1,3-glucan localization was investigated with a primary mouse antibody specific to β-1,3-glucan in conjunction with the anti-mouse secondary antibody CF^488^. β-1,3-Glucan-specific labeling was rarely observed around tubular hyphae developed on the surface of CMs, with labeling mostly observed at hyphal breakage points (Fig. 8A). To label β-1,3-glucan present on the surface of infection-like structures formed inside CMs, sandpaper had to be used to create antibody entry points. In doing so, we observed an intense but patchy labeling at the periphery of the infection-like structures (Fig. 8A). A similar patchy distribution was also observed on tubular hyphae developed on the surface of apple leaves and etiolated hypocotyls (Fig. 8B; Fig. S6B). In contrast, β-1,3-glucan was detected around the entire cell periphery of *V. inaequalis* in cross sections of infected apple leaves (Fig. 8C). Interestingly, labeling of β-1,3-glucan was not observed on young subcuticular infection structures developed in leaves (Fig. 8B) or hypocotyls (Fig. S6B). Instead, labeling of β-1,3-glucan could only be observed on mature stromata that had ruptured the apple cuticle upon sporulation (Fig. 8B).

As *V. inaequalis* has two putative α-1,3-glucan synthase genes and, of these, *ViAGS2* is upregulated during early host colonization (Fig. 5), we attempted to label α-1,3-glucan using the primary antibody MOPC-104E in conjunction with the secondary antibody anti-mouse CF^488^. Labeling of α-1,3-glucan was observed on tubular hyphae developed on the surface of CMs (Fig. 8D), but not on tubular hyphae developed on the surface of apple tissue (data not shown). Likewise, no labeling of α-1,3-glucan was observed on the surface of infection-like structures formed in culture or on the subcuticular infection structures developed *in planta* (data not shown).

## DISCUSSION

Even though the cell wall plays a crucial role in viability and pathogenesis (20, 30, 62), few studies have focused on the cell wall of plant-pathogenic fungi. Here, we examined the cell wall carbohydrate composition of sporulating tubular hyphae from *V. inaequalis* developed on the surface of cellophane membranes (CMs) using glycosidic linkage analysis. As observed in other fungi, the most abundant glucosyl linkages found in *V. inaequalis* were 1,3-Glc, with the proportion identified similar to that found in the subcuticular plant pathogen *R. secalis* (63) (Table 1). Glycosidic linkage analysis does not allow discrimination between α- and β-linkages. However, using CLSM, we were able to show that α-1,3-glucan was present on surface hyphae developed in culture. Therefore, a fraction of the identified 1,3-Glc could be from α-1,3-glucan, which coats the surface of the *V. inaequalis* cell wall.

**TABLE 1:** Carbohydrate composition of the Venturia inaequalis cell wall, determined in this study using glycosidic linkage analysis, compared to other fungal species for which carbohydrate composition is known^*a*^

Carbohydrate	% of carbohydrate in:					
	*V. inaequalis*	*B. graminis* f. sp. *hordei*	*R. secalis*	*N. crassa*	*A. fumigatus*	*S. cerevisiae*
t-Man	18.9	1.7	3.1	1.5	0.1	15.7
t-Glc	4.3	5.7	13.9	13	0.3	5.5
t-Gal	7.3	2.2	0	1	2.2	0
3-Glc	18.5	35.6	11.6	54	43.3	26.9
2-Man	12.4	4.3	4.3	3	2.4	7.9
6-Man	1.7	3.8	4.4	0	1.2	1.2
6-Glc	1.7	2	0.5	0	0.3	0.7
4-Gal	1.1	6.8	0	0	0	0
4-Glc	11.7	6.3	17.2	4	14.2	0
2,3-Man	2.2	0	0.9	0	0	0
3,4-Glc	1.8	2.1	0.5	0	0.3	0.7
2,3-Glc	1.2	7.5	1.5	0	0	7
3,6-Glc	3.5	1.6	2.7	5	1.6	2
3,6-Man	2.3	0.6	0.6	0	0	0.4
2,6-Hxp	7.8	0	0	0	0	0
4,6-Glc	1.5	2.3	0.5	0	0	0
4,6-Hxp	2.2	0	0	0	0	0
1,4-GlcNAc	3.8	8.7	6.3	10	17.65	0.6
3,6-Gal	0.0	0	0	0	0	0

a This comparison was made for the filamentous fungi Blumeria graminis f. sp. *hordei* (72), Rhynchosporium secalis (63), Neurospora crassa (73), Aspergillus fumigatus (101) (alkali-insoluble fraction of the cell wall), and the yeast Saccharomyces cerevisiae (71). This table was extracted and modified from reference 72. Gal, galactose; Glc, glucose; GlcNAc, *N*-acetylglucosamine; Man, mannose; Hxp, hexopyranose.

Surprisingly, α-1,3-glucan could not be detected on the surface of *V. inaequalis* infection-like structures developed inside CMs or on the subcuticular infection structures developed *in planta*, even though the putative α-1,3-glucan synthase gene *ViAGS2* was upregulated during early host colonization. However, it should be noted that, while upregulated, the expression level of *ViAGS2* was low (see Fig. S4 and Supplemental File 5 in the supplemental material). The lack of α-1,3-glucan labeling *in planta* could indicate that this carbohydrate is not label-accessible on the cell wall surface during host colonization. However, it is more plausible that this is due to the problem we encountered with antibody penetration inside host tissue. Interestingly, in some human fungal pathogens, such as Histoplasma capsulatum and Aspergillus fumigatus, as well as the fungal plant pathogen Magnaporthe oryzae, α-1,3-glucan accumulates on the surface of the fungal cell wall to conceal cell-wall-derived MAMPs and, thus, prevents the induction of host defense responses during host infection (41–44, 64). Additionally, in A. fumigatus, α-1,3-glucan is important for hyphal and conidial aggregation (65, 66). With this research in mind, additional experiments are now needed to investigate whether α-1,3-glucan is present on the surface of subcuticular infection structures produced by *V. inaequalis in planta*.

Another abundant glucosyl linkage identified as part of our glycosidic linkage analysis was 1,4-Glc. It cannot be ruled out that a portion of this 1,4-Glc arose from cellulose contamination from the CM itself or an intracellular form of a glycogen/starch-like polysaccharide that copurifies with the cell wall during sample preparation. Nevertheless, the small amount of 1,4,6-Glc identified suggests that not all of the 1,4-Glc originated from glycogen/starch-like polymers. Notably, there is very little evidence for the presence of β-1,4-linked glucosyl residues in fungal cell walls, although, interestingly, these residues were recently reported in the cell wall of A. fumigatus by solid-state nuclear magnetic resonance (47, 67). Hence, it cannot be ruled out that a small fraction of the reported 1,4-Glc from *V. inaequalis* originates from β-1,4-glucan. Another more likely possibility is that a portion of the identified 1,4-Glc forms part of nigeran, a polymer commonly found in Ascomycota fungal cell walls that are composed of α-1,3- and α-1,4-glucans (68).

The presence of 1,3,6-Glc suggests that a fraction of the identified 1,3-Glc is branched. A set of *GH17* and *GH72* genes found to be highly expressed in culture likely encode the enzymes responsible for the presence of 1,3,6-linked glucosyl residues in the *V. inaequalis* cell wall, as these enzymes are putative β-1,3-glucan transglycosylases involved in cross-linking β-1,3-glucans through β-1,6-linkages (28, 53, 54, 69). Interestingly, during mid to late infection, *V. inaequalis* was observed to downregulate a gene encoding a KRE6 enzyme, *ViKRE6*, which is putatively associated with β-1,6-glucan biosynthesis (55, 56, 70). We suggest that, as reported in Colletotrichum graminicola (70), *V. inaequalis* downregulates *ViKRE6* to reduce surface-exposed β-1,3-6-glucans that would otherwise elicit plant immune responses. As a starting point to investigate this possibility, future work could focus on the expression dynamics of this gene during host infection using promoter-fluorophore fusions in conjunction with CLSM.

The glycosidic linkage analysis also revealed a relatively large proportion of Man (~37%) and Gal (~8%) residues in the cell wall of *V. inaequalis*, with the majority being terminal. Due to this terminal nature, we cannot conclude from which polymer these residues are derived. The large amount of t-Man (18.9%), however, is similar to the amount of t-Man reported in S. cerevisiae (15.7%) (71) and drastically more than those of other filamentous fungi (1 to 3%) (63, 72, 73).

Following glycosidic linkage analysis, we attempted to investigate the presence and location of β-1,3- and branched β-1,3-6-glucans on the fungal cell wall surface using a specific antibody that labels β-1,3-glucan. Regarding the subcuticular infection structures developed inside the host, we only observed strong labeling on the surface of mature stromata that were rupturing through the apple cuticle as part of the sporulation process. It is unclear whether these subcuticular infection structures were labeled because the mature stromata were more accessible to the β-1,3-glucan antibody or because the β-1,3-glucan is masked during early host colonization and is only surface-exposed upon sporulation.

The glycosidic linkage analysis also revealed that the cell wall of sporulating tubular hyphae from *V. inaequalis* is comprised of 3.8% chitin, which is relatively low for a filamentous fungus. Indeed, chitin usually makes up around 10 to 15% of the fungal cell wall (18, 25, 27) (Table 1). This result is in line with the labeling profile of chitin present on the surface of tubular hyphae formed on CMs using the WGA^488^ probe and calcofluor white, where chitin was mostly restricted to septa. Transcriptomic and proteomic data obtained in our study suggest that ViCHS3b, a class III CHS enzyme, might be responsible for the bulk of chitin biosynthesis in *V. inaequalis* tubular hyphae.

Interestingly, on the plant surface, chitin was exclusively observed on tubular hyphae and appressoria, while on the subcuticular infection structures produced after host penetration, chitin was found to be restricted to the septa. Furthermore, genes encoding CHSs and some chitinases were downregulated during early host colonization. These findings suggest that, in addition to transcriptionally regulating chitin production, *V. inaequalis* also restricts the amount of chitin it exposes on its surface to prevent activation of chitin-triggered host defenses. Another possibility is that *V. inaequalis* masks chitin on the cell wall surface through the secretion of chitin-binding effectors, similar to that shown for the Avr4 effector protein of Fulvia fulva (74). Alternatively, *V. inaequalis* may produce chitin-binding effectors that sequester chitin oligomers during the process of cell wall remodeling to prevent their detection by the apple immune system. Notably, during *in planta* host colonization, the lysin motif (LysM) domain-containing effector candidate, ViEcp6, which has sequence similarity to the chitin-binding Ecp6 effector of *F. fulva* (75, 76), was found to be upregulated (Fig. S4) (48) and had proteomic support in culture (Supplemental Files 3 and 4). Although Ecp6 from *F. fulva* is known to sequester chitin oligomers to evade host defense responses in tomato (77), other LysM domain-containing effectors, such as Mg3LysM from Zymoseptoria tritici, have been shown to protect the hyphal cell wall from hydrolysis by plant chitinases (78). It is therefore possible that ViEcp6 plays a role in chitin protection and/or sequestration during subcuticular host colonization.

Another possibility could be that *V. inaequalis* deacetylates chitin to chitosan during host colonization to avoid activation of plant defenses, as reported for other plant-associated fungal pathogens (33, 35, 79, 80) and endophytes (34). In line with this, we observed that runner hyphae and stromata developed in culture and *in planta* were completely covered with chitosan. This indicates that chitosan is the main surface-exposed carbohydrate present on these structures. Interestingly, chitosan was also observed on tubular hyphae developed on the surface of hypocotyls and chitosan production could be induced during growth on the surface of CMs coated with apple wax. These results suggest that chitosan induction is triggered by a plant-derived cue present in the apple wax. Additionally, chitosan labeling was observed at the periphery of infection-like structures developed inside CMs, indicating that another trigger, such as pressure, might be involved. It is important to note here, however, that although the OGA^488^ probe is specific for chitosan (60), binding to the positively charged groups of the carbohydrate, it cannot be ruled out that it also binds to positively charged proteins present on the surface of these structures.

In addition to preventing activation of the plant immune system, chitosan has also been reported to be important for cell adhesion and morphogenesis in fungi (80–83). Therefore, chitosan may play other functions in *V. inaequalis*. The deacetylation of chitin to chitosan is catalyzed by CDAs, and an inspection of the *V. inaequalis* genome revealed that this fungus has eight predicted CDA-encoding genes, three of which (*ViCDA1*, *ViCDA4*, and *ViCDA7*) are predicted to be functional. One of these, *ViCDA1*, encodes a protein with a predicted N-terminal signal peptide and is constitutively expressed in culture and during host colonization. Secreted CDAs are usually assumed to deacetylate chitin to chitosan to prevent activation of the plant immune system (50, 84). Therefore, it seems plausible that ViCDA1 is the main enzyme responsible for deacetylating chitin to chitosan on the surface of subcuticular infection structures produced by *V. inaequalis*. In any case, the recently developed CRISPR-Cas9 method for reverse genetics in this fungus (85) will serve to investigate the putative role of ViCDA1 and other CDAs in *V. inaequalis*.

In conclusion, we have detailed, for the first time, the cell wall carbohydrate composition of *V. inaequalis* during growth in culture. Furthermore, by assessing the expression profile of genes putatively associated with cell wall biogenesis, as well as by monitoring the localization of cell-surface-associated carbohydrates using CLSM, we have also provided new insights into how this fungus differentiates and protects its subcuticular infection structures during host colonization. Importantly, as the first study of its kind for a subcuticular pathogen, our research provides a foundation for understanding not only how this class of plant-pathogenic fungi causes disease, but also how they can potentially be controlled.

## MATERIALS AND METHODS

### *V. inaequalis* isolate.

*V. inaequalis* isolate MNH120, also known as ICMP 13258 and Vi1 (76, 86), derived from a monospore culture, was used in this study.

### Preparation of fungal material for glycosidic linkage analysis and proteomics.

Fungal material for glycosidic linkage analysis and proteomics was prepared by inoculating s100 μL of 10^5^ to 10^6^ conidia from *V. inaequalis* on cellophane membranes (CMs) (Waugh Rubber Bands, Wellington, New Zealand) overlaying PDA (Scharlab, S.L., Senmanat, Spain) and culturing for 5 days at 22°C under a 16-h/8-h light/dark cycle. Following culturing, fungal biomass, comprising a mixture of tubular hyphae and asexual conidia, was harvested from the surface of the CMs using an L-shaped spreader, washed three times by centrifugation at 4,000 × *g* for 15 min, frozen at −20°C, freeze-dried, and then ground to a fine powder in liquid nitrogen.

### Glycosidic linkage analysis.

Cell wall material from *V. inaequalis* grown in culture on the surface of CMs overlaying PDA at 5 dpi was prepared in triplicate (i.e., as three technical replicates) using a previously described protocol (87). The cell wall preparations were subjected to permethylation and gas chromatography-electron impact mass spectrometry (GC-EI MS) (87). Partially methylated alditol acetate (PMAA) derivatives were separated and analyzed by gas chromatography (GC) on an SP-2380 capillary column (30 m by 0.25 mm [inner diameter]) (Supelco) using an HP-6890 GC system with an HP-5973 electron impact mass spectrometer (EI MS) as a detector (Agilent Technologies, CA, USA). The temperature was programmed to increase from 180°C to 230°C at a rate of 1.5°C/min. The mass spectra of the fragments obtained from the PMAA derivatives were compared with reference derivatives.

### Proteomic analysis.

Approximately 10 mg of powdered fungal material from *V. inaequalis* grown in culture on the surface of CMs overlaying PDA at 5 dpi in triplicate (i.e., as three technical replicates) was boiled in SDS buffer (75 mM Tris-HCl buffer [pH 6.8] containing 3% [wt/vol] SDS, 100 mM dithiothreitol [DTT], 15% [wt/vol] glycerol, and 0.002% bromophenol blue) for 5 min at 95°C. Insoluble material was then removed by centrifugation at 14,000 × *g* for 10 min, and the supernatant was loaded on a 12% Mini-Protean TGX SDS-PAGE system (Bio-Rad; CA, USA). After staining with Coomassie blue (Thermo Fisher Scientific, MA, USA), the gel lane was cut into 10 bands and the proteins were subjected to in-gel digestion with trypsin (Promega, Madison, WI, USA) as previously described (88).

Peptide analysis was performed by reverse-phase liquid chromatography electrospray ionization–tandem mass spectrometry (LC-ESI-MS/MS) using a nanoACQUITY ultraperformance liquid chromatography system coupled to a quadrupole time of flight (Q-TOF) mass spectrometer (Xevo Q-TOF; Waters, MA, USA). The in-gel tryptic peptides were resuspended in 0.1% trifluoroacetic acid (TFA) and loaded on a Waters Symmetry C_18_ trap column (180 μm by 20 mm, 5 μm) that was then washed with 0.1% (vol/vol) formic acid at 10 μL/min for 5 min. The samples eluted from the trap column were separated on a Waters C_18_ analytical column (75 μm by 150 mm, 1.7 μm) at 250 nL/min using 0.1% formic acid as solvent A and 0.1% formic acid in acetonitrile as solvent B in a stepwise gradient: 0 to 10% B (0 to 5 min), 10 to 30% B (5 to 70 min), 30 to 40% B (70 to 72 min), 40 to 90% B (72 to 75 min), 90% B (75 to 80 min), and 90 to 0.1% B (80 to 85 min). The eluting peptides were sprayed in the mass spectrometer (capillary and cone voltages set to 2.1 kV and 45 V, respectively), and MS/MS spectra were acquired using automated data-directed switching between the MS and MS/MS modes using the instrument’s software (MassLynx v.4.0 SP4). The five most abundant signals of a survey scan (400- to 1,300-*m/z* range, 1-s scan time) were selected by charge state, and collision energy was applied accordingly for sequential MS/MS fragmentation scanning (100- to 1,800-*m/z* range, 1-s scan time).

The resulting MS raw data files were processed using Mascot Distiller (v.2.4.3.2) (Matrix Science, London, United Kingdom), and the resulting files were submitted to a local Mascot server (v2.3.1; Matrix Science) using the *Venturia* protein database (25,153 sequences) (48). The following settings were used for database search: trypsin-specific digestion with two missed cleavages, ethanolated cysteine as fixed and oxidized methionine as variable modifications, peptide tolerance of 200 ppm, and fragment tolerance of 0.2 Da. Only those peptides with Mascot scores exceeding the threshold for statistical significance (*P* < 0.05) were retained. The peak list files generated from the mass spectrometry raw data have been deposited in the MassIVE database (accession no. MSV000090342).

### Plant growth conditions and infection of apple material.

Open-pollinated Malus x domestica cultivar “Royal Gala” apple seeds (Hawke’s Bay, New Zealand) were germinated at 4°C in moist vermiculite containing 100 mg/mL Thiram fungicide (Kiwicare Corporation, Ltd., Christchurch, New Zealand) for ~2 months in the dark. Once germinated, apple seedlings were planted in Dalton’s premium potting mix (Daltons, Matamata, New Zealand) and grown under a 16-h/8-h light/dark cycle with a Philips SON-T AGRO 400 sodium lamp at 20°C with ambient humidity for 4 to 6 weeks.

For the growth of etiolated hypocotyls, a fast-germination method was used. Seeds were sterilized with 5% ethanol for 5 min, rinsed five times with sterile Milli-Q water, and soaked in Milli-Q water overnight. The next morning, the testa of the apple seeds were peeled away with sterile forceps until the underlying white embryo was uncovered. Then, the white embryo was placed in Murashige and Skoog (MS) agar medium (2. 25 g/L MS medium [Sigma-Aldrich, Castle Hill, Australia], 10 g/L agar at pH 5.8 [KOH]) inside 50-mL Falcon tubes, with the tip of the radical submerged in the agar. Once the apple seeds had germinated and the cotyledons were fully expanded, the seedlings were transferred to potting mix soil and grown at 20°C with ambient humidity in the dark for 2 weeks. *V. inaequalis* infection of detached apple leaves and hypocotyls was performed as described by Rocafort et al. (48) and Shiller et al. (89), respectively.

### Extraction of apple wax and coating of cellophane membranes.

Approximately 10 fruit from apple cultivar “Royal Gala,” acquired from a local supermarket in New Zealand, were used for wax extraction. To extract the wax, each apple was dipped a total of five times, each for 10 s, in 200 mL chloroform contained within a glass beaker. Following this step, the chloroform in the beaker was evaporated off at room temperature (RT) until the extracted wax had completely dried out. To coat the CMs with apple wax, a Kimwipe (Kimtech, Milsons Point, Australia) was dipped in chloroform, wiped over the surface of the wax in the beaker, and then transferred by wiping onto the surface of CMs until a homogeneous white coating was observed. Coating of the CMs with apple wax was performed under sterile conditions as much as possible, with coated CMs subsequently subjected to 15 min of UV exposure to ensure sterility.

### Confocal laser scanning microscopy.

Cross-sections of detached apple leaves infected with *V. inaequalis* at 7 dpi were prepared for labeling as described previously (9). Briefly, leaf tissue was fixed in paraformaldehyde and 2.5% (vol/vol) glutaraldehyde in 0.1 M phosphate buffer (pH 7.2) and then embedded in LR White resin (London Resin, Reading, United Kingdom), with samples subsequently cross-sectioned from the resin-embedded material.

All the other samples (non-cross-sectioned), CMs associated with *V. inaequalis* at 6 to 7 dpi, as well as detached apple leaves and etiolated hypocotyls infected with *V. inaequalis* at 7 dpi were fixed in 95% ethanol and stored at 4°C until required. Additionally, to enhance the penetrability of the carbohydrate-specific probes and antibodies used for labeling (described below), *in planta* samples were macerated with 10% KOH at RT for 4 h. For α-1,3-glucan and β-1,3-glucan labeling, non-cross-sectioned CM samples were gently scratched with sandpaper (200 grit) to generate entry points for the antibody. All samples were washed 3 times with 1 mL phosphate-buffered saline (PBS) buffer (pH 7.4) prior to labeling.

To facilitate the visualization of subcuticular infection structures produced by *V. inaequalis* in apple hypocotyls, fungal nuclei were stained with 0.002% (wt/vol) propidium iodide (PI) in PBS buffer (pH 7.4) containing 0.02% Tween 20. For the labeling of chitin and chitosan, carbohydrate-specific probes were used. More specifically, for WGA^488^ labeling of chitin, samples were vacuum infiltrated in the dark for 30 min in a solution containing 0.1 mg/mL of WGA^488^ probe and 0.02% Tween 20 in PBS buffer (pH 7.4). For calcofluor white labeling of chitin, samples were incubated in 300 mM sodium hydroxide (NaOH) for 1 h prior to incubation with 0.01% calcofluor white (Sigma-Aldrich) in PBS (pH 7.4). For OGA^488^ labeling of chitosan, samples were vacuum infiltrated in the dark for 30 min with a solution of 0.1% (wt/vol) OGA^488^ and 0.02% Tween 20 in PBS buffer (pH 7.4). For this purpose, a 1-mg/mL stock solution of OGA^488^ in 0.1 M sodium acetate buffer (pH 4.9) was kindly provided by Jozef Mravec from the University of Copenhagen (60). In all cases, the probe used for labeling was vacuum infiltrated into samples for 20 min, twice, in the dark.

For the labeling of β-1,3-glucan in cross-sectioned and non-cross-sectioned samples, and α-1,3-glucan in non-cross-sectioned samples, carbohydrate-specific antibodies were used. More specifically, samples were first washed three times with 1 mL PBS buffer (pH 7.4) and blocked with 3% bovine serum albumin (BSA) (Gibco, MD, USA) in PBS buffer for 1 h at RT in a rotatory shaker (John Morris Scientific, Palmerston North, New Zealand). Then, samples were washed with 1 mL PBS buffer three times and vacuum infiltrated for 20 min with 0.1 mg/mL of primary mouse anti-1,3-β-glucan antibody (Biosupplies, Sydney, Australia) for β-1,3-glucan labeling or 0.1 mg/mL mouse MOPC-104E primary antibody (Sigma-Aldrich) for α-1,3-glucan labeling and then incubated overnight with shaking (30 rpm) at 4°C. The next day, samples were washed three times with 1 mL PBS buffer (pH 7.4) and vacuum infiltrated for 20 min with 0.1 mg/mL anti-mouse CF^488^ secondary antibody (Biotum, CA, USA) in PBS buffer (pH 7.4) and incubated with shaking at 30 rpm at RT in the dark. Finally, samples were washed three times with 1 mL PBS buffer.

CLSM was performed on labeled samples using a Leica SP5 DM6000B confocal microscope (488-nm argon and 405-nm UV laser) (Leica Microsystems, Manheim, Germany), with images produced using ImageJ 1.x software (NIH) (90). Here, multiple optical sections (z-stacks) were projected into a single image as maximum- or average-intensity projections. PI was excited at 561 nm using a DPSS laser with an emission spectrum of 561 to 600 nm. WGA^488^, OGA^488^ and CF^488^ were excited using a 488-nm argon laser (power, ~30%) with an emission spectrum of 498 to 551 nm. Calcofluor white was excited using a 405-nm UV laser with an emission spectrum of 445 to 455 nm. For all samples, an appropriate nonstained control was performed (Fig. S7).

### Annotation of fungal cell wall enzymes.

The predicted *V. inaequalis* isolate MNH120 protein catalogue from Rocafort et al. (48) (10.5281/zenodo.6233646 available at https://zenodo.org/record/6233646#.ZCMtPcrMJPY) was used in this study. Proteins that were putatively associated with fungal cell wall biogenesis were identified based on CAZyme annotation and KEGG classification, and decisions about the potential classification of enzymes involved in cell wall biosynthesis were further assessed by InterProscan scan annotation (in conjunction with the Pfam, HAMAP, MOBIDB, PIRSF, PROSITE, and SUPERFAMILY tools). Here, all InterProScan and CAZyme annotations, as well as KEGG classifications, were provided by Rocafort et al. (48).

To identify CDAs, proteins with a CE4 CAZyme annotation or with the polysaccharide deacetylase domain (PF01522) were annotated as CDAs. To further investigate other putative CDAs in the genome, a blastP (91) search was performed against the *V. inaequalis* MNH120 genome (76) using the protein sequence of CDAs from pathogens whose activity has been experimentally shown: *P. graminis* (GMQ_17027) (92), Colletotrichum lindemuthianum (ClCDA) (AAT68493) (50), *Pestalotiopsis* sp. (PesCDA) (KY024221) (84), and M. oryzae (MGG_12939, MGG_14966, MGG_09159, MGG_04172, MGG_08774, MGG_01868, MGG_08356, MGG_05023, MGG_04704, and MGG_03461) (80). Although the enzymatic activity of the M. oryzae CDAs has not been experimentally shown, their function as potential CDAs has been reported by gene deletion studies (31, 33, 80). The E value cutoff used for all blastp searches was 1E−02. To investigate whether the putative CDAs had the conserved amino acid residues required for catalytic activity and metal binding, an alignment was performed between all of the predicted *V. inaequalis* CDAs and the functionally characterized CDAs mentioned above, ClCDA and PesCDA (93), using the MUSCLE plug-in of Geneious v.9.0.5.

To classify CHSs, the InterProScan domain annotation from Rocafort et al. (48) was used and an alignment of all *V. inaequalis* CHSs was performed using the MUSCLE plugin of Geneious v.9.0.5 (93). The CHSs from the Aspergillus nidulans (94), Neurospora crassa (95), Botrytis cinerea (96), and Ustilago maydis were included in this alignment as a reference. To determine if the predicted CHSs of *V. inaequalis* had the conserved motifs required for CHS activity, a protein sequence alignment was performed with the functionally characterized CHS from N. crassa (95) as a reference.

### Gene expression analysis.

Preexisting *V. inaequalis* gene expression data from Rocafort et al. (48) (GEO series accession no. GSE198244) were used for the gene expression analysis, and differentially expressed genes were identified using DESeq2 package v.1.32.0 (97). Genes up- or downregulated at one or more *in planta* infection time points, relative to growth in culture, with a log_2_ fold change of ±1.5 and a *P* value of 0.01 were considered differentially expressed. Volcano plots were generated using ggplots2 v.3.3.5 (98), while gene expression heat maps were generated using Complexheatmap v.2.9.1 (99).

### Real-time quantitative PCR.

A total of 400 ng of RNA, extracted as part of our previous RNA-seq study (48), was used for cDNA synthesis and was generated with a Qiagen QuantiTect reverse transcription kit according to the manufacturer’s instructions. RT-qPCR was performed using a Bioline SensiFASTTM SYBR No-ROX kit in conjunction with the primers listed in Supplemental File 5. For each reaction, 1 μL of undiluted cDNA was added to 9 μL of PCR mixture following the manufacturer’s instructions. The cycling conditions were 40 cycles of 5 s at 95°C, 10 s at 60°C, and 20 s at 72°C. Relative expression of target genes was calculated with the threshold cycle (2^–ΔΔ^*^CT^*) method (100) using the geometric mean of two *V. inaequalis* housekeeping genes, coding for β-tubulin (*g10951*) and 60S ribosomal protein (*g3362*), as a reference (9). Fold change was calculated relative to expression levels in culture.

## References

[1] Bowen JK, Mesarich CH, Bus VG, Beresford RM, Plummer KM, Templeton MD. 2011. *Venturia inaequalis*: the causal agent of apple scab. Mol Plant Pathol 12:105–122. doi:10.1111/j.1364-3703.2010.00656.x. 21199562 PMC6640350

[2] Carisse O, Bernier J. 2002. Effect of environmental factors on growth, pycnidial production and spore germination of *Microsphaeropsis* isolates with biocontrol potential against apple scab. Mycol Res 106:1455–1462. doi:10.1017/S0953756202006858.

[3] Jha G, Thakur K, Thakur P. 2009. The *Venturia* apple pathosystem: pathogenicity mechanisms and plant defense responses. J Biomed Biotechnol 2009:680160. doi:10.1155/2009/680160. 20150969 PMC2817808

[4] MacHardy WE. 1996. Apple scab: biology, epidemiology, and management. The American Phytopathological Society, Saint Paul, MN.

[5] Le Cam B, Sargent D, Gouzy J, Amselem J, Bellanger M-N, Bouchez O, Brown S, Caffier V, De Gracia M, Debuchy R, Duvaux L, Payen T, Sannier M, Shiller J, Collemare J, Lemaire C. 2019. Population genome sequencing of the scab fungal species *Venturia inaequalis*, *Venturia pirina*, *Venturia aucupariae* and *Venturia asperata*. G3 (Bethesda) 9:2405–2414. doi:10.1534/g3.119.400047. 31253647 PMC6686934

[6] Manktelow D, Beresford R, Batchelor T, Walker J. 1996. Use patterns and economics of fungicides for disease control in New Zealand apples. Acta Hortic 422:187–192.

[7] Patocchi A, Wehrli A, Dubuis PH, Auwerkerken A, Leida C, Cipriani G, Passey T, Staples M, Didelot F, Philion V, Peil A, Laszakovits H, Rühmer T, Boeck K, Baniulis D, Strasser K, Vávra R, Guerra W, Masny S, Ruess F, Le Berre F, Nybom H, Tartarini S, Spornberger A, Pikunova A, Bus VGM. 2020. Ten years of VINQUEST: first insight for breeding new apple cultivars with durable apple scab resistance. Plant Dis 104:2074–2081. doi:10.1094/PDIS-11-19-2473-SR. 32525450

[8] Gessler C, Stumm D. 1984. Infection and stroma formation by *Venturia inaequalis* on apple leaves with different degrees of susceptibility to scab. J Phytopathol 110:119–126. doi:10.1111/j.1439-0434.1984.tb03399.x.

[9] Kucheryava N, Bowen JK, Sutherland PW, Conolly JJ, Mesarich CH, Rikkerink EH, Kemen E, Plummer KM, Hahn M, Templeton MD. 2008. Two novel *Venturia inaequalis* genes induced upon morphogenetic differentiation during infection and *in vitro* growth on cellophane. Fungal Genet Biol 45:1329–1339. doi:10.1016/j.fgb.2008.07.010. 18692586

[10] Nusbaum CJ, Keitt GW. 1938. A cytological study of host-parasite relations of *Venturia inaequalis* on apple leaves. J Agric Res 56:595–618.

[11] Caffier V, Le Cam B, Expert P, Tellier M, Devaux M, Giraud M, Chevalier M. 2012. A new scab-like disease on apple caused by the formerly saprotrophic fungus *Venturia asperata*. Plant Pathol 61:915–924. doi:10.1111/j.1365-3059.2011.02583.x.

[12] Latham AJ, Rushing AE. 1988. Development of *Cladosporium caryigenum* in pecan leaves. Phytopathology 78:1104–1108. doi:10.1094/Phyto-78-1104.

[13] Lanza B, Ragnelli AM, Priore M, Aimola P. 2017. Morphological and histochemical investigation of the response of *Olea europaea* leaves to fungal attack by *Spilocaea oleagina*. Plant Pathol 66:1239–1247. doi:10.1111/ppa.12671.

[14] Hunter GC, Zeil-Rolfe I, Jourdan M, Morin L. 2021. Exploring the host range and infection process of *Venturia paralias* isolated from *Euphorbia paralias* in France. Eur J Plant Pathol 159:811–823. doi:10.1007/s10658-021-02204-z.

[15] Blechert O, Debener T. 2005. Morphological characterization of the interaction between *Diplocarpon rosae* and various rose species. Plant Pathol 54:82–90. doi:10.1111/j.1365-3059.2005.01118.x.

[16] Zhan J, Fitt BDL, Pinnschmidt HO, Oxley SJP, Newton AC. 2008. Resistance, epidemiology and sustainable management of *Rhynchosporium secalis* populations on barley. Plant Pathol 57:1–14. doi:10.1111/j.1365-3059.2007.01691.x.

[17] Linsell KJ, Keiper FJ, Forgan A, Oldach KH. 2011. New insights into the infection process of *Rhynchosporium secalis* in barley using GFP. Fungal Genet Biol 48:124–131. doi:10.1016/j.fgb.2010.10.001. 20955811

[18] Free SJ. 2013. Fungal cell wall organization and biosynthesis. Adv Genet 81:33–82. doi:10.1016/B978-0-12-407677-8.00002-6. 23419716

[19] Latgé J-P. 2007. The cell wall: a carbohydrate armour for the fungal cell. Mol Microbiol 66:279–290. doi:10.1111/j.1365-2958.2007.05872.x. 17854405

[20] Sánchez-Vallet A, Mesters JR, Thomma BP. 2015. The battle for chitin recognition in plant-microbe interactions. FEMS Microbiol Rev 39:171–183. doi:10.1093/femsre/fuu003. 25725011

[21] Zhang J, Zhou J-M. 2010. Plant immunity triggered by microbial molecular signatures. Mol Plant 3:783–793. doi:10.1093/mp/ssq035. 20713980

[22] Saijo Y, Loo E, Yasuda S. 2018. Pattern recognition receptors and signaling in plant-microbe interactions. Plant J 93:592–613. doi:10.1111/tpj.13808. 29266555

[23] Gonçalves IR, Brouillet S, Soulié M-C, Gribaldo S, Sirven C, Charron N, Boccara M, Choquer M. 2016. Genome-wide analyses of chitin synthases identify horizontal gene transfers towards bacteria and allow a robust and unifying classification into fungi. BMC Evol Biol 16:252. doi:10.1186/s12862-016-0815-9. 27881071 PMC5122149

[24] Gow NAR, Latge JP, Munro CA. 2017. The fungal cell wall: structure, biosynthesis, and function. Microbiol Spectr doi:10.1128/microbiolspec.FUNK-0035-2016. PMC11687499 28513415

[25] Osherov N, Yarden O. 2010. The cell wall of filamentous fungi, p 224–237. *In* Borkovich KA, Ebbole DJ (ed), Cellular and molecular biology of filamentous fungi. ASM Press, Washington, DC.

[26] Mouyna I, Henry C, Doering TL, Latgé J-P. 2004. Gene silencing with RNA interference in the human pathogenic fungus *Aspergillus fumigatus*. FEMS Microbiol Lett 237:317–324. doi:10.1016/j.femsle.2004.06.048. 15321679

[27] Garcia-Rubio R, de Oliveira HC, Rivera J, Trevijano-Contador N. 2019. The fungal cell wall: *Candida*, *Cryptococcus*, and *Aspergillus* species. Front Microbiol 10:2993. doi:10.3389/fmicb.2019.02993. 31993032 PMC6962315

[28] Mouyna I, Hartl L, Latgé J. 2013. β-1,3-Glucan modifying enzymes in *Aspergillus fumigatus*. Front Microbiol 4:81. doi:10.3389/fmicb.2013.00081. 23616783 PMC3627985

[29] Rovenich H, Zuccaro A, Thomma BPHJ. 2016. Convergent evolution of filamentous microbes towards evasion of glycan-triggered immunity. New Phytol 212:896–901. doi:10.1111/nph.14064. 27329426

[30] de Oliveira Silva A, Aliyeva-Schnorr L, Wirsel SGR, Deising HB. 2022. Pathogenesis-related cell wall biogenesis with emphasis on the maize anthracnose fungus *Colletotrichum graminicola*. Plants 11:849. doi:10.3390/plants11070849. 35406829 PMC9003368

[31] Geoghegan I, Steinberg G, Gurr S. 2017. The role of the fungal cell wall in the infection of plants. Trends Microbiol 25:957–967. doi:10.1016/j.tim.2017.05.015. 28641930

[32] Gao F, Zhang B-S, Zhao J-H, Huang J-F, Jia P-S, Wang S, Zhang J, Zhou J-M, Guo H-S. 2019. Deacetylation of chitin oligomers increases virulence in soil-borne fungal pathogens. Nat Plants 5:1167–1176. doi:10.1038/s41477-019-0527-4. 31636399

[33] Geoghegan IA, Gurr SJ. 2017. Investigating chitin deacetylation and chitosan hydrolysis during vegetative growth in *Magnaporthe oryzae*. Cell Microbiol 19:e12743. doi:10.1111/cmi.12743. 28371146 PMC5573952

[34] Noorifar N, Savoian MS, Ram A, Lukito Y, Hassing B, Weikert TW, Moerschbacher BM, Scott B. 2021. Chitin deacetylases are required for *Epichloë festucae* endophytic cell wall remodeling during establishment of a mutualistic symbiotic interaction with *Lolium perenne*. Mol Plant Microbe Interact 34:1181–1192. doi:10.1094/MPMI-12-20-0347-R. 34058838

[35] Rizzi YS, Happel P, Lenz S, Urs MJ, Bonin M, Cord-Landwehr S, Singh R, Moerschbacher BM, Kahmann R. 2021. Chitosan and chitin deacetylase activity are necessary for development and virulence of *Ustilago maydis*. mBio 12:e03419-20. doi:10.1128/mBio.03419-20. 33653886 PMC8092297

[36] Iriti M, Faoro F. 2009. Chitosan as a MAMP, searching for a PRR. Plant Signal Behav 4:66–68. doi:10.4161/psb.4.1.7408. 19704712 PMC2634077

[37] Gubaeva E, Gubaev A, Melcher RLJ, Cord-Landwehr S, Singh R, El Gueddari NE, Moerschbacher BM. 2018. ‘Slipped sandwich’ model for chitin and chitosan perception in *Arabidopsis*. Mol Plant Microbe Interact 31:1145–1153. doi:10.1094/MPMI-04-18-0098-R. 29787346

[38] Vander P, Vårum KM, Domard A, Eddine El Gueddari N, Moerschbacher BM. 1998. Comparison of the ability of partially N-acetylated chitosans and chitooligosaccharides to elicit resistance reactions in wheat leaves. Plant Physiol 118:1353–1359. doi:10.1104/pp.118.4.1353. 9847109 PMC34751

[39] Ride JP, Barber MS. 1990. Purification and characterization of multiple forms of endochitinase from wheat leaves. Plant Sci 71:185–197. doi:10.1016/0168-9452(90)90008-C.

[40] Lopez-Moya F, Suarez-Fernandez M, Lopez-Llorca LV. 2019. Molecular mechanisms of chitosan interactions with fungi and plants. Int J Mol Sci 20:332. doi:10.3390/ijms20020332. 30650540 PMC6359256

[41] Rappleye CA, Engle JT, Goldman WE. 2004. RNA interference in *Histoplasma capsulatum* demonstrates a role for α-(1,3)-glucan in virulence. Mol Microbiol 53:153–165. doi:10.1111/j.1365-2958.2004.04131.x. 15225311

[42] Rappleye CA, Eissenberg LG, Goldman WE. 2007. *Histoplasma capsulatum* alpha-(1,3)-glucan blocks innate immune recognition by the beta-glucan receptor. Proc Natl Acad Sci USA 104:1366–1370. doi:10.1073/pnas.0609848104. 17227865 PMC1783108

[43] Fujikawa T, Sakaguchi A, Nishizawa Y, Kouzai Y, Minami E, Yano S, Koga H, Meshi T, Nishimura M. 2012. Surface α-1,3-glucan facilitates fungal stealth infection by interfering with innate immunity in plants. PLoS Pathog 8:e1002882. doi:10.1371/journal.ppat.1002882. 22927818 PMC3426526

[44] Fujikawa T, Kuga Y, Yano S, Yoshimi A, Tachiki T, Abe K, Nishimura M. 2009. Dynamics of cell wall components of *Magnaporthe grisea* during infectious structure development. Mol Microbiol 73:553–570. doi:10.1111/j.1365-2958.2009.06786.x. 19602150

[45] Ibe C, Munro CA. 2021. Fungal cell wall: an underexploited target for antifungal therapies. PLoS Pathog 17:e1009470. doi:10.1371/journal.ppat.1009470. 33886695 PMC8061829

[46] Jaworski C, Wang L. 1965. Gross cell wall composition of *V. inaequalis* cell wall. Phytopathology 55:401–403.

[47] Ruiz-Herrera J, Ortiz-Castellanos L. 2019. Cell wall glucans of fungi. A review. Cell Surf 5:100022. doi:10.1016/j.tcsw.2019.100022. 32743138 PMC7389562

[48] Rocafort M, Bowen JK, Hassing B, Cox MP, McGreal B, de la Rosa S, Plummer KM, Bradshaw RE, Mesarich CH. 2022. The *Venturia inaequalis* effector repertoire is dominated by expanded families with predicted structural similarity, but unrelated sequence, to avirulence proteins from other plant-pathogenic fungi. BMC Biol 20:246. doi:10.1186/s12915-022-01442-9. 36329441 PMC9632046

[49] Mandel AM, Galgiani JN, Scott K, Orbach MJ. 2006. *Coccidioides posadasii* contains single chitin synthase genes corresponding to classes I to VII. Fungal Genet Biol 43:775–788. doi:10.1016/j.fgb.2006.05.005. 16857399

[50] Blair DE, Hekmat O, Schüttelkopf AW, Shrestha B, Tokuyasu K, Withers SG, van Aalten DM. 2006. Structure and mechanism of chitin deacetylase from the fungal pathogen *Colletotrichum lindemuthianum*. Biochemistry 45:9416–9426. doi:10.1021/bi0606694. 16878976

[51] Hekmat O, Tokuyasu K, Withers SG. 2003. Subsite structure of the endo-type chitin deacetylase from a deuteromycete, *Colletotrichum lindemuthianum*: an investigation using steady-state kinetic analysis and MS. Biochemistry J 374:369–380. doi:10.1042/bj20030204. PMC1223603 12775215

[52] Ramazzina I, Cendron L, Folli C, Berni R, Monteverdi D, Zanotti G, Percudani R. 2008. Logical identification of an allantoinase analog (*puuE*) recruited from polysaccharide deacetylases. J Biol Chem 283:23295–23304. doi:10.1074/jbc.M801195200. 18550550

[53] Mouyna I, Hartland RP, Fontaine T, Diaquin M, Simenel C, Delepierre M, Henrissat B, Latgé J. 1998. A 1,3-beta-glucanosyltransferase isolated from the cell wall of *Aspergillus fumigatus* is a homologue of the yeast *Bgl2p*. Microbiology 144:3171–3180. 10.1099/00221287-144-11-3171 9846753

[54] Patel P, Free SJ. 2022. Characterization of *Neurospora crassa* GH16, GH17, and GH72 gene families of cell wall crosslinking enzymes. Cell Surf 8:100073. doi:10.1016/j.tcsw.2022.100073. 35079668 PMC8777122

[55] Gilbert NM, Donlin MJ, Gerik KJ, Specht CA, Djordjevic JT, Wilson CF, Sorrell TC, Lodge JK. 2010. *KRE* genes are required for beta-1,6-glucan synthesis, maintenance of capsule architecture and cell wall protein anchoring in *Cryptococcus neoformans*. Mol Microbiol 76:517–534. doi:10.1111/j.1365-2958.2010.07119.x. 20384682 PMC2969852

[56] Levinson JN, Shahinian S, Sdicu A-M, Tessier DC, Bussey H. 2002. Functional, comparative and cell biological analysis of *Saccharomyces cerevisiae Kre5p*. Yeast 19:1243–1259. doi:10.1002/yea.908. 12271460

[57] Mouyna I, Monod M, Fontaine T, Henrissat B, Léchenne B, Latgé JP. 2000. Identification of the catalytic residues of the first family of beta(1–3)glucanosyltransferases identified in fungi. Biochem J 347:741–747. doi:10.1042/bj3470741. 10769178 PMC1221011

[58] Ragni E, Fontaine T, Gissi C, Latgè JP, Popolo L. 2007. The Gas family of proteins of *Saccharomyces cerevisiae*: characterization and evolutionary analysis. Yeast 24:297–308. doi:10.1002/yea.1473. 17397106

[59] Nicholson RL, Van Scoyoc S, Williams EB, Kuc J. 1973. Etiolated apple hypocotyls: a useful host tissue in apple scab research. Phytopathology 63:363–366. doi:10.1094/Phyto-63-363.

[60] Mravec J, Kračun SK, Rydahl MG, Westereng B, Miart F, Clausen MH, Fangel JU, Daugaard M, Van Cutsem P, De Fine Licht HH, Höfte H, Malinovsky FG, Domozych DS, Willats WGT. 2014. Tracking developmentally regulated post-synthetic processing of homogalacturonan and chitin using reciprocal oligosaccharide probes. Development 141:4841–4850. doi:10.1242/dev.113365. 25395456

[61] Fesel PH, Zuccaro A. 2016. β-Glucan: crucial component of the fungal cell wall and elusive MAMP in plants. Fungal Genet Biol 90:53–60. doi:10.1016/j.fgb.2015.12.004. 26688467

[62] Verdín J, Sánchez-León E, Rico-Ramírez AM, Martínez-Núñez L, Fajardo-Somera RA, Riquelme M. 2019. Off the wall: the rhyme and reason of *Neurospora crassa* hyphal morphogenesis. Cell Surf 5:100020. doi:10.1016/j.tcsw.2019.100020. 32743136 PMC7389182

[63] Pettolino F, Sasaki I, Turbic A, Wilson SM, Bacic A, Hrmova M, Fincher GB. 2009. Hyphal cell walls from the plant pathogen *Rhynchosporium secalis* contain (1,3/1,6)-β-d-glucans, galacto- and rhamnomannans, (1,3;1,4)-β-d-glucans and chitin. FEBS J 276:3698–3709. doi:10.1111/j.1742-4658.2009.07086.x. 19496815

[64] Beauvais A, Bozza S, Kniemeyer O, Formosa C, Balloy V, Henry C, Roberson RW, Dague E, Chignard M, Brakhage AA, Romani L, Latgé J-P. 2013. Deletion of the α-(1,3)-glucan synthase genes induces a restructuring of the conidial cell wall responsible for the avirulence of *Aspergillus fumigatus*. PLoS Pathog 9:e1003716. doi:10.1371/journal.ppat.1003716. 24244155 PMC3828178

[65] Yoshimi A, Miyazawa K, Abe K. 2017. Function and biosynthesis of cell wall α-1,3-glucan in fungi. J Fungi (Basel) 3:63. doi:10.3390/jof3040063. 29371579 PMC5753165

[66] Fontaine T, Beauvais A, Loussert C, Thevenard B, Fulgsang CC, Ohno N, Clavaud C, Prevost M-C, Latgé J-P. 2010. Cell wall α1-3glucans induce the aggregation of germinating conidia of *Aspergillus fumigatus*. Fungal Genet Biol 47:707–712. doi:10.1016/j.fgb.2010.04.006. 20447463

[67] Kang X, Kirui A, Muszyński A, Widanage MCD, Chen A, Azadi P, Wang P, Mentink-Vigier F, Wang T. 2018. Molecular architecture of fungal cell walls revealed by solid-state NMR. Nat Commun 9:2747. doi:10.1038/s41467-018-05199-0. 30013106 PMC6048167

[68] Ruiz-Herrera J. 1991. Fungal cell wall: structure, synthesis, and assembly. CRC Press, Boca Raton, FL.

[69] Gastebois A, Mouyna I, Simenel C, Clavaud C, Coddeville B, Delepierre M, Latgé J-P, Fontaine T. 2010. Characterization of a new β(1–3)-glucan branching activity of *Aspergillus fumigatus*. J Biol Chem 285:2386–2396. doi:10.1074/jbc.M109.077545. 19948732 PMC2807297

[70] Oliveira-Garcia E, Deising HB. 2016. Attenuation of PAMP-triggered immunity in maize requires down-regulation of the key β-1,6-glucan synthesis genes *KRE5* and *KRE6* in biotrophic hyphae of *Colletotrichum graminicola*. Plant J 87:355–375. doi:10.1111/tpj.13205. 27144995

[71] Fleet GH. 1985. Composition and structure of yeast cell walls, p 24–56. *In* McGinnis MR (ed), Current topics in medical mycology. Springer, New York, NY. 10.1007/978-1-4613-9547-8_2 3916769

[72] Pham TAT, Kyriacou BA, Schwerdt JG, Shirley NJ, Xing X, Bulone V, Little A. 2019. Composition and biosynthetic machinery of the *Blumeria graminis* f. sp. *hordei* conidia cell wall. Cell Surf 5:100029. doi:10.1016/j.tcsw.2019.100029. 32743145 PMC7388969

[73] Mélida H, Sain D, Stajich JE, Bulone V. 2015. Deciphering the uniqueness of Mucoromycotina cell walls by combining biochemical and phylogenomic approaches. Environ Microbiol 17:1649–1662. doi:10.1111/1462-2920.12601. 25143134

[74] van den Burg HA, Harrison SJ, Joosten MH, Vervoort J, de Wit PJ. 2006. *Cladosporium fulvum* Avr4 protects fungal cell walls against hydrolysis by plant chitinases accumulating during infection. Mol Plant Microbe Interact 19:1420–1430. doi:10.1094/MPMI-19-1420. 17153926

[75] Bolton MD, Van Esse HP, Vossen JH, De Jonge R, Stergiopoulos I, Stulemeijer IJE, Van Den Berg GCM, Borrás-Hidalgo O, Dekker HL, De Koster CG, De Wit PJGM, Joosten MHAJ, Thomma BPHJ. 2008. The novel *Cladosporium fulvum* lysin motif effector Ecp6 is a virulence factor with orthologues in other fungal species. Mol Microbiol 69:119–136. doi:10.1111/j.1365-2958.2008.06270.x. 18452583

[76] Deng CH, Plummer KM, Jones DAB, Mesarich CH, Shiller J, Taranto AP, Robinson AJ, Kastner P, Hall NE, Templeton MD, Bowen JK. 2017. Comparative analysis of the predicted secretomes of Rosaceae scab pathogens *Venturia inaequalis* and *V. pirina* reveals expanded effector families and putative determinants of host range. BMC Genomics 18:339. doi:10.1186/s12864-017-3699-1. 28464870 PMC5412055

[77] de Jonge R, Peter van Esse H, Kombrink A, Shinya T, Desaki Y, Bours R, van der Krol S, Shibuya N, Joosten Matthieu HAJ, Thomma Bart PHJ. 2010. Conserved fungal LysM effector Ecp6 prevents chitin-triggered immunity in plants. Science 329:953–955. doi:10.1126/science.1190859. 20724636

[78] Marshall R, Kombrink A, Motteram J, Loza-Reyes E, Lucas J, Hammond-Kosack KE, Thomma BPHJ, Rudd JJ. 2011. Analysis of two in planta expressed LysM effector homologs from the fungus *Mycosphaerella graminicola* reveals novel functional properties and varying contributions to virulence on wheat. Plant Physiol 156:756–769. doi:10.1104/pp.111.176347. 21467214 PMC3177273

[79] El Gueddari NE, Rauchhaus U, Moerschbacher BM, Deising HB. 2002. Developmentally regulated conversion of surface-exposed chitin to chitosan in cell walls of plant pathogenic fungi. New Phytol 156:103–112. doi:10.1046/j.1469-8137.2002.00487.x.

[80] Geoghegan IA, Gurr SJ. 2016. Chitosan mediates germling adhesion in *Magnaporthe oryzae* and is required for surface sensing and germling morphogenesis. PLoS Pathog 12:e1005703. doi:10.1371/journal.ppat.1005703. 27315248 PMC4912089

[81] Kamakura T, Yamaguchi S, Saitoh K, Teraoka T, Yamaguchi I. 2002. A novel gene, *CBP1*, encoding a putative extracellular chitin-binding protein, may play an important role in the hydrophobic surface sensing of *Magnaporthe grisea* during appressorium differentiation. Mol Plant Microbe Interact 15:437–444. doi:10.1094/MPMI.2002.15.5.437. 12036274

[82] Kuroki M, Okauchi K, Yoshida S, Ohno Y, Murata S, Nakajima Y, Nozaka A, Tanaka N, Nakajima M, Taguchi H, Saitoh K-I, Teraoka T, Narukawa M, Kamakura T. 2017. Chitin-deacetylase activity induces appressorium differentiation in the rice blast fungus *Magnaporthe oryzae*. Sci Rep 7:9697. doi:10.1038/s41598-017-10322-0. 28852173 PMC5575296

[83] Banks IR, Specht CA, Donlin MJ, Gerik KJ, Levitz SM, Lodge JK. 2005. A chitin synthase and its regulator protein are critical for chitosan production and growth of the fungal pathogen *Cryptococcus neoformans*. Eukaryot Cell 4:1902–1912. doi:10.1128/EC.4.11.1902-1912.2005. 16278457 PMC1287864

[84] Cord-Landwehr S, Melcher RLJ, Kolkenbrock S, Moerschbacher BM. 2016. A chitin deacetylase from the endophytic fungus *Pestalotiopsis* sp. efficiently inactivates the elicitor activity of chitin oligomers in rice cells. Sci Rep 6:38018. doi:10.1038/srep38018. 27901067 PMC5128826

[85] Rocafort M, Arshed S, Hudson D, Sidhu JS, Bowen JK, Plummer KM, Bradshaw RE, Johnson RD, Johnson LJ, Mesarich CH. 2022. CRISPR-Cas9 gene editing and rapid detection of gene-edited mutants using high-resolution melting in the apple scab fungus, *Venturia inaequalis*. Fungal Biol 126:35–46. doi:10.1016/j.funbio.2021.10.001. 34930557

[86] Stehmann C, Pennycook S, Plummer KM. 2001. Molecular identification of a sexual interloper: the pear pathogen, *Venturia pirina*, has sex on apple. Phytopathology 91:633–641. doi:10.1094/PHYTO.2001.91.7.633. 18942992

[87] Mélida H, Sandoval-Sierra JV, Diéguez-Uribeondo J, Bulone V. 2013. Analyses of extracellular carbohydrates in oomycetes unveil the existence of three different cell wall types. Eukaryot Cell 12:194–203. doi:10.1128/EC.00288-12. 23204192 PMC3571302

[88] Leijon F, Melzer M, Zhou Q, Srivastava V, Bulone V. 2018. Proteomic analysis of plasmodesmata from populus cell suspension cultures in relation with callose biosynthesis. Front Plant Sci 9:1681. doi:10.3389/fpls.2018.01681. 30510561 PMC6252348

[89] Shiller J, Van de Wouw AP, Taranto AP, Bowen JK, Dubois D, Robinson A, Deng CH, Plummer KM. 2015. A large family of *AvrLm6*-like genes in the apple and pear scab pathogens, *Venturia inaequalis* and *Venturia pirina*. Front Plant Sci 6:980. doi:10.3389/fpls.2015.00980. 26635823 PMC4646964

[90] Schneider CA, Rasband WS, Eliceiri KW. 2012. NIH Image to ImageJ: 25 years of image analysis. Nat Methods 9:671–675. doi:10.1038/nmeth.2089. 22930834 PMC5554542

[91] Altschul SF, Madden TL, Schäffer AA, Zhang J, Zhang Z, Miller W, Lipman DJ. 1997. Gapped BLAST and PSI-BLAST: a new generation of protein database search programs. Nucleic Acids Res 25:3389–3402. doi:10.1093/nar/25.17.3389. 9254694 PMC146917

[92] Naqvi S, Cord-Landwehr S, Singh R, Bernard F, Kolkenbrock S, El Gueddari NE, Moerschbacher BM. 2016. A recombinant fungal chitin deacetylase produces fully defined chitosan oligomers with novel patterns of acetylation. Appl Environ Microbiol 82:6645–6655. doi:10.1128/AEM.01961-16. 27590819 PMC5086558

[93] Kearse M, Moir R, Wilson A, Stones-Havas S, Cheung M, Sturrock S, Buxton S, Cooper A, Markowitz S, Duran C, Thierer T, Ashton B, Meintjes P, Drummond A. 2012. Geneious Basic: an integrated and extendable desktop software platform for the organization and analysis of sequence data. Bioinformatics 28:1647–1649. doi:10.1093/bioinformatics/bts199. 22543367 PMC3371832

[94] Mouyna I, Dellière S, Beauvais A, Gravelat F, Snarr B, Lehoux M, Zacharias C, Sun Y, de Jesus Carrion S, Pearlman E, Sheppard DC, Latgé J-P. 2020. What are the functions of chitin deacetylases in *Aspergillus fumigatus*? Front Cell Infect Microbiol 10:28. doi:10.3389/fcimb.2020.00028. 32117802 PMC7016196

[95] Fajardo-Somera RA, Jöhnk B, Bayram Ö, Valerius O, Braus GH, Riquelme M. 2015. Dissecting the function of the different chitin synthases in vegetative growth and sexual development in *Neurospora crassa*. Fungal Genet Biol 75:30–45. doi:10.1016/j.fgb.2015.01.002. 25596036

[96] Choquer M, Boccara M, Gonçalves IR, Soulié MC, Vidal-Cros A. 2004. Survey of the *Botrytis cinerea* chitin synthase multigenic family through the analysis of six euascomycetes genomes. Eur J Biochem 271:2153–2164. doi:10.1111/j.1432-1033.2004.04135.x. 15153106

[97] Love MI, Huber W, Anders S. 2014. Moderated estimation of fold change and dispersion for RNA-seq data with DESeq2. Genome Biol 15:550. doi:10.1186/s13059-014-0550-8. 25516281 PMC4302049

[98] Wickham H. 2016. ggplot2: elegant graphics for data analysis. Springer-Verlag, New York, NY.

[99] Gu Z, Eils R, Schlesner M. 2016. Complex heatmaps reveal patterns and correlations in multidimensional genomic data. Bioinformatics 32:2847–2849. doi:10.1093/bioinformatics/btw313. 27207943

[100] Livak KJ, Schmittgen TD. 2001. Analysis of relative gene expression data using real-time quantitative PCR and the 2^−ΔΔCT^ method. Methods 25:402–408. doi:10.1006/meth.2001.1262. 11846609

[101] Fontaine T, Simenel C, Dubreucq G, Adam O, Delepierre M, Lemoine J, Vorgias CE, Diaquin M, Latgé J-P. 2000. Molecular organization of the alkali-insoluble fraction of *Aspergillus fumigatus* cell wall. J Biol Chem 275:27594–27607. doi:10.1074/jbc.M909975199. 10869365

