# Supplementary material for: Cell Wall Carbohydrate Dynamics during the Differentiation of Infection Structures by the Apple Scab Fungus, Venturia inaequalis
Source: Microbiol Spectr. 2023 Apr 11;11(3):e04219-22. doi: 10.1128/spectrum.04219-22 (PMC10269774; doi:10.1128/spectrum.04219-22)
Supplement: Supplemental file 4 — Supplemental material. Download spectrum.04219-22-s0004.pdf, PDF file, 1.7 MB [file spectrum.04219-22-s0004.pdf]

## Supplementary Information

**Supplementary file 1: *Venturia inaequalis* genes that encode enzymes putatively associated with fungal cell wall biogenesis.** Enzymes were classified according to their predicted roles in cell wall biogenesis, as based on predicted 'Kyoto Encyclopedia of Genes and Genomes' (KEGG) and InterProScan annotations. CBM, carbohydrate-binding module; CDA, chitin deacetylase; CE, carbohydrate esterase; CHS, chitin synthase; GH, glycoside hydrolase; GT, glycosyl transferase; PL, polysaccharide lyase. * Only three CE4 proteins were predicted to be a carbohydrate-active enzyme (CAZYme) using dbCan2.

**Table S1. *Venturia inaequalis* carbohydrate-active enzymes (CAZymes) putatively involved in fungal cell wall biogenesis and modification.** Enzymes were classified according to their predicted roles in cell wall biogenesis, as based on predicted 'Kyoto Encyclopedia of Genes and Genomes' (KEGG) and InterProScan annotations. CBM, carbohydrate-binding molecule; CE, carbohydrate esterase; GH, glycoside hydrolase; GPI, glycosylphosphatidylinositol; GT, glycosyl transferase; PL, polysaccharide lyase; UDP, uridine diphosphate. * Only three CE4 proteins were predicted to be a carbohydrate-active enzyme (CAZYme) using dbCan2.

| Enzyme | CAZyme family | Copy number |
|---|---|---|
| **CHITIN METABOLISM** | | |
| Chitin synthase | GT2 | 8 |
| Chitinase | GH18 | 5 |
| Hexosaminadase | GH20 | 1 |
| β-N-acetylhexosaminidase | GH3 | 1 |
| Chitin deacetylase | CE4 | 8* |
| **GLUCAN METABOLISM** | | |
| β-1,3-glucan synthase | GT48 | 1 |
| α-1,3-glucan synthase | GH13+GT15 | 2 |
| 1,3-β-glucanosyltransferase | GH72 | 6 |
| β-glycanase | GH16 | 13 |
| 1,3-β-glucanase | GH128, GH152, GH64, GH81 | 8 |
| β-glucosidase | GH1, GH132, GH17, GH3, GH5, GH55 | 29 |
| 1,4-α-glucan branching enzyme | CBM48+GH13_8 | 1 |
| α-amylase | GH13 | 7 |
| alpha-1,6-glucohydrolase | GH13 | 2 |
| glucan 1,4-alpha-glucosidase | GH15 | 2 |
| α-glucosidase | GH31, GH71 | 6 |

| | | |
|---|---|---|
| **MANNOSE METABOLISM** | | |
| α-1,2-mannosyltransferase | GT15, GT4, GT71 | 7 |
| α-1,3-mannosyltransferase | GT69 | 7 |
| α-1,6-mannosyltransferase | GT22, GT34 | 6 |
| α-1,3/α-1,6-mannosyltransferase | GT4 | 1 |
| GPI-mannosyltransferase | GT22, GT50, GT76 | 4 |
| β-1,4-mannosyltransferase | GT33 | 1 |
| Dolichyl-phosphate-mannose-protein mannosyltransferase | GT39 | 3 |
| Mannan polymerase complex MNN9/ANP1 | GT62 | 2 |
| α-1,2-mannosidase | GH92, GH47 | 13 |
| Mannan endo-1,6-α-mannosidase | GH76 | 8 |
| Mannosyl-oligosaccharide glucosidase | GH63, GH31 | 3 |
| α-mannosidase | GH38 | 1 |
| β-mannosidase | GH2 | 1 |
| Mannosyl-glycoprotein endo- β -N-acetylglucosaminidase | GH85 | 1 |

| | | |
|---|---|---|
| **GALACTOSE METABOLISM** | | |
| Inositol 3-alpha-Galactosyltransferase | GT8 | 1 |
| α-galactosidase | GH27 | 6 |
| β-galactosidase | GH2 | 1 |
| α-1,4-galactosaminogalactan hydrolase | GH135 | 4 |
| α-1,4-polygalactosaminidase | GH114 | 9 |
| N-acetylgalactosamine deacetylase | CE18+CBM87 | 4 |
| Endo- β -1,6-galactanase | GH5 | 1 |
| UDP-glucose 4-epimerase | - | 3 |

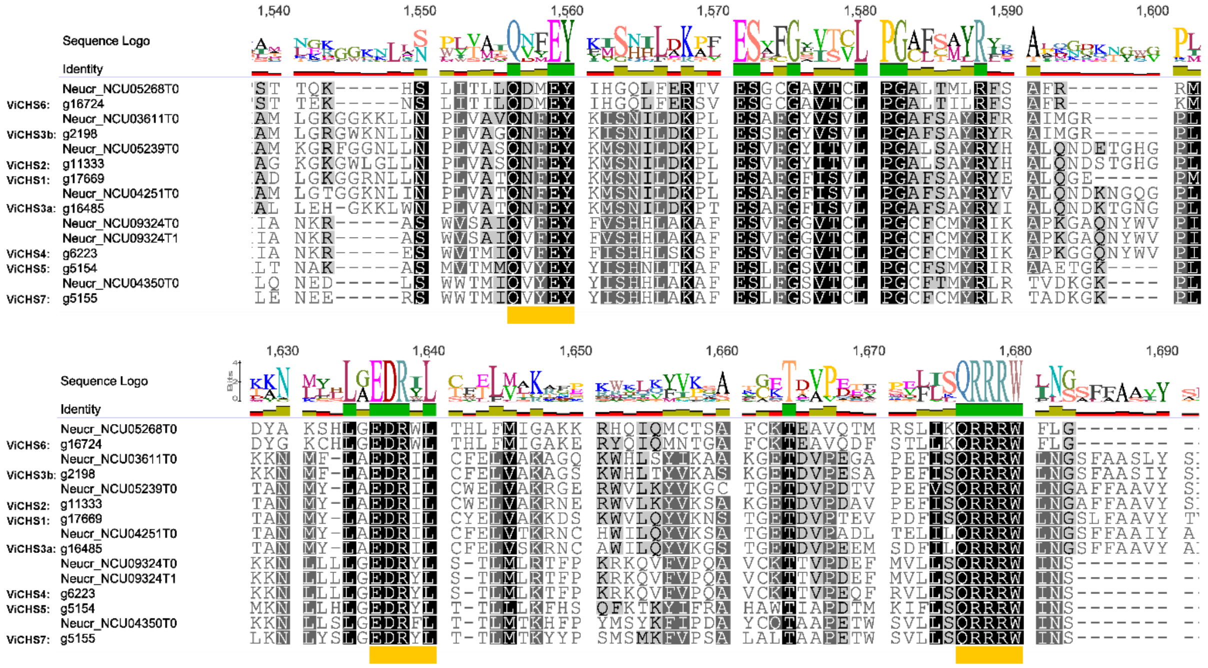


**Figure S1. Multiple sequence alignment of putative chitin synthase (CHS) proteins from *Venturia inaequalis*.** The CHS proteins from *Neurospora crassa* (Neucr) were used as reference for alignment. Alignment generated using the MUSCLE plugin of Geneious v9.0.5 in conjunction with full-length protein sequences. The conserved motifs for catalytic activity are highlighted under the alignment with orange boxes. Amino acids are coloured based on similarity, with the most similar amino acids coloured black.

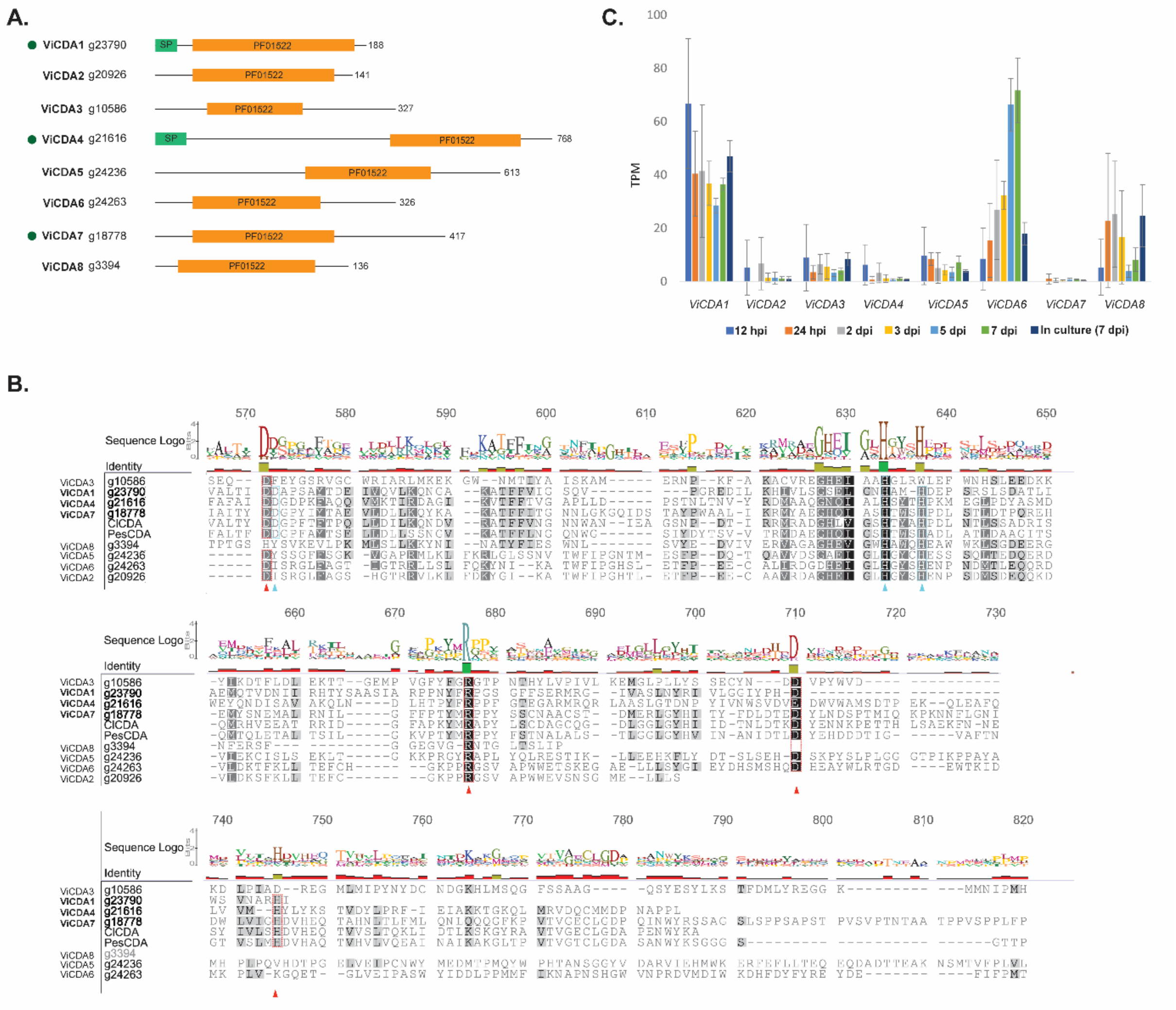


**Figure S2. Predicted chitin deacetylases (CDAs) of *Venturia inaequalis*. A.** Domain organization of putative CDA proteins from *V. inaequalis*. Only ViCDA1, ViCDA4 and ViCDA7 display all conserved amino acid residues required for catalytic activity and metal binding and, therefore, are likely to be active (labelled with green circles). Protein lengths are shown. SP, Signal peptide; PF01522, Protein family (Pfam) polysaccharide deacetylase domain. **B**. Multiple sequence alignment of putative CDA proteins from *V. inaequalis*. CDA proteins from *Colletotrichum lindemuthianum* (ClCDA) and *Pestalotiopsis* sp. (PesCDA) were used as references for alignment. Alignment generated using the MUSCLE plugin of Geneious v9.0.5 in conjunction with full-length protein sequences. The conserved amino acids for catalytic activity are highlighted with red triangles under the alignment and with dashed red boxes. Conserved amino acids for metal binding are highlighted with cyan triangles under the alignment and with dashed cyan boxes. Amino acids are coloured based on similarity, with the most similar amino acids coloured black. **C.** Expression of the eight predicted *V. inaequalis CDA* genes during growth of the fungus *in planta* (12 hours post-inoculation [hpi] to 7 days post-inoculation [dpi]) and in culture on the surface of cellophane membranes (CMs) (7 dpi). Gene expression is shown as transcripts per million (TPM). Error bars represent standard deviation across four biological replicates.

**Supplementary file 2: List of genes encoding putative cell wall biogenesis proteins from *Venturia inaequalis* that are up- or down-regulated at one or more *in planta* time points during infection of apple leaves (12 hours post-inoculation [hpi] to 7 days post-inoculation [dpi]), relative to growth of the fungus in culture on the surface of cellophane membranes (CMs) overlaying potato dextrose agar (7 dpi).**

**Supplementary file 3: List of proteins identified by mass spectrometry from *Venturia inaequalis* grown on the surface of cellophane membranes overlaying potato dextrose agar at 5 days post-inoculation, and short-list of proteins putatively associated with fungal cell wall biogenesis from this list.** Only peptides exceeding the threshold for statistical significance ($p < 0.05$) were selected.

**Supplementary file 4**: **Peptide coverage of proteins identified by mass spectrometry from *Venturia inaequalis* grown on the surface of cellophane membranes overlaying potato dextrose agar at 5 days post-inoculation that are putatively associated with fungal cell wall biogenesis.**

**Peptide detected**

Modification: **X**, oxidation; X, ethanolyl

### Glucose metabolism: α-glucan

>g23025: CBM48+GH13_8

MQDAMASTSATVGDGKSNIPNDGTGVVTLDPWLEPFKDSLRSRFAKAQSWIK**TINDTEGGLEK**FSRGYEK**FGFNV**
**LPNNDVVYR**EWAPNALR**AYLIGDFNNWNR**DSHQMTKDEYGAFDITIPAVNGQPAIPHDSKIKISLVIPNDGHRAE
R**IPAWITRVTQDLNVSPVYDARFWNPPANEK**YIFKNKPPPQPKSVRIYEAHVGISSWEGKVATYKEFTRNVLPRI
KNDGYNVIQLMAIMEHAYYASFGYQINSFFAASSRYGLPDELKELVDTAHGMGITVLLDVVHSHASKNVLDGLNT
FDGSDHQYFHEGAKGRHELWDSRLFNYGNHEVLR**FLLSNLRFWMEEYQFDGFR**FDGVTSMMYTHHGIGTGFSGGY
HEYFGSNVDEEAVAYMMIANELLHTLYPRVITVAEDVSGMPALCVELALGGIGFDYR**LAMAIPDLYIK**WLKEKED
IEWDMGALCFTLTNRRHGEKTIAYAESHDQALVGDKSLMMWLCDAELYTNMSILSVETPVITRGLSLHKMIRLIT
HGLGGEGYLNFEGNEFGHPEWLDFPREGNGNSFHYARR**QFNLADDQLLR**YRFLNDFDSKMQWTEEKYGWLHSPQA
YVSLKNETDKVIVFERAGLLWIFNFHPNQSYTDYR**VGVEEAGTYRVVINTDSK**SFGGHGNIKEETRFFTTDFAWN
GRK**NFVQVYIPSR**TAMVLALEGTL

>g5073: GH31

MPNMNDYKFPSEPLANPDSTITGKNYRFTLIDDKLLRYEWSADGKFEDRASTFAINRKFPKPEFRIEETDSQLEI
FTPRYHLIYDKQRFSENGLVVQFTSKQSEWGGEWRYGYGPQQNLGGTARTLDGVDGRCDMGAGILSRVGYAALDD
TGSMLFDGEGFVGTRLEGDRIDGYLFAYGFDLRGAMKSFYAVSGSQPVVPRWCLGNWWSRYHDYTADEYLELMDD
FKKKEIPMSVAVIDMDWHIVHGDDVPHVGWTGYTWDKKLFPDPGAFTKALHDRKLKTTLNDHPHAGVWHHEDSYE
EMAKVLGHDTTNKKPILFDPTSQEFMHAFLHVLHRNLEKEGCDFWWIDWQQGSHSRVPGLDPLWLLNHFHFIDQE
QVKGKSEALIFSRYAGPGSHRYPVGFSGDAFATWPSLEFQPEFTATASNIGYGWWSHDIGGHLPGFRDDECATR**W**
**IQLGVFSPILR**LHSTRSRWMSKEPWLYRSECETAMRDAMQLRHRLVPYIYSENANTPTSSMPLVQPIYWNFPERN
IAYKFPNQFYFGSELVVSPVVTPRDPRTNLAKTKVWVPPARHVDLLAGLIYDGDREIDVYRSLNDVPVLAK**EGTI**

**IPLDGAK**VPANGCGNPDAFEVLVVVGQDGNYNILEDTRDDAKPVETESPRSILIDYKQAEGKLSVDGNGRAWTFR
**FISFTDDLSNIK**VSSSDAEVSVTSTPTPSLTVTIPTTTDKITIELGSNPQLSVLDNTKSISDILVNYQININIKD
EIWKALTAEQATITKMGRLLSLGLEEALVGPLVEFLVADSRSINGGMTKEKQYAGVIAQGD

>g748: ViAGS1 GH13_22+GT5

MHVFVWVVLTLCARVFGAAYEASEVDFNLNQNQSATHPLDYYGEWSNHTYNKSPSNWR**FPFYTLFLDR**LANGDPS
NDNANGTLFEQDILSNQFRHGGDVAGLLDSLDYIQGMGMKGLYIAGSPFMNQPWTADSYSPVDLTILDHHFGTID
TWRTVITAIHARGMYVVLDNTFATMGDLFGFDGYLNTTTPFSLAEHKSQWKGQRRYLDFDIGEEYNTTCAYPRFW
NETGYRIDSIYTDKMKGCYNSEFDQYGDTEAFGVYPDFQRQLTKFASVQDRLREWHPPVLEKINHFSCMVIKMLD
IDGFRFDKATQITVDAQAGFGQYMRNCAKSVGKDNFFMPGEITGGNTFGSIYLGRGRQPDMLPENATVATKMTNN
SNSKYFIRDQGMGALDAAAFHYTIYRSLTRFLGMDGNLEAGYDANGGTWTEAWNTMLLTNDMVNENSGIFDPRHM
FGVTNQDVFRWPAISQGTERMILGMFVTAIHLPGIPLVTFGEEQAFYVLDSTADNYIYGRQPMTASIAWQMHGCY
SLGSSQYYQMPLNDSRNGCKDDTVSYDHRDPSHPVRNILKHMYFLREQFPVLNDGWYLQDLSNQTHDVVLPGSSG
KPTETGIWSVVRKEFTGSQTLSSNQTIWFVYHNVNETTTYTFDCNSDKTSFLSAFDKDTTAKNLMYPFDEITLGS
TTTTKANTGCVSNITMKPYEFKVYVPK**AAFVTALPMITK**FLPGHDVRVKSSVSPGEQETVDVEIQFSAVMSCDAV
TSSILLNSTVEDLRIAQIKSGSVKCSTLNSTTTRYVGDLPSAWTWKATLENVSNGIHAITVKNASRTDGTATNSV
DRFIFRVGQDDNPMVFPHTANYTRALIHTNGSSKDLYISHKAAGADKWRYSLTWGSSWSNWENYYGGNSTLQQQN
WTGTSLQKWDGTHIMAQYWSRLSGSSAVIQHADLGREGQPPRRFPHLFAQGPYNQYGFDSGLKNKVELTKDGSWD
WHFMSEWPDTVQLNVWGMNPDGQPDAAFVYGDIDADGILDRLPPSSLSNLAINVTNGPPSPWLAWKLSIDDGTLR
YTLLPVGNRWLQLALFMLMAFIPLITACLAVWAYMRGFYEVKFNEVGVSEK**TGFMALPLALR**RGRGMKKLAQFAS
EDNLTNMDSAIPFSTAVAGLSEKRRTVLIATLEYDIDDWNIKIKIGGLGVMAQLMGKNLEHQDLIWVVPCVGGVD
YPPATAAEPMEVIILGEKYDIQVQVHQLR**NITYVLLDAPIFR**AQSKTEPYPPRMDDLDSAVYYSAWNQCIARAIQ
RYPVDLYHINDYHGACAPLYLLPETIPCCLSLHNAEFQGLWPMRTPKERAEVCSVFNLSEKTVAQYVQFGSVFNL
LHAGASYLRVHQKGVGAVGVSNKYGKRSWARYPIFWGLKAIGKLPNPDPTDTEAWDKQLPKEGDIHVDPTYEAGR
GELKRQAQEWAGLEQRADAELFVFVGRWSVQKGVDLIADVFPAVLEHHEKVQLICIGPVIDLYGKFAALKLGKMM
EKYPGRVYSKPEFTALPPYIFSGAEFALIPSRDEPFGLVAVEFGRKGALGVGARVGGLGQMPGWWYTVESTTSAH
LLKQFKESIDTALASSQETRAMMRARSAKQRFPVQQWKEDLGILQSKCIKLHDQEVEKHGGRLTRHHDSGAPAPP
IVTLENNELRSVSSPTTGSAVEGPSSGGLKRTLSLGVRQGPGHRASGDGDGENHPENIPEEEEYIVEHGDQTAGE
EGNPDSLPLPPWPIRGPGSRFSTASAMSAVSFDPQRASTATEHPSPVPSPAFLRPDRDGAESPGYYEPDSLVPPS
PGFYQQPDANRSSLLSLNLVVGEKKDFKLQKVDPFFTDSNMEYFHAFESKLGDLNGKNSEHDLCIEEYLIKSERT
WFGKFRDARLGRTTEKGIAGSRVTSRAPSPSPSTYRFPNDSYSEEDENDKTPLRDEFLLGDDYKPPTGLKKHAQM
KVGDWPIYTLLLAIGQIISANSYQVTLLTGSVGQAASKLYVVASIYLVASIGWWVLFRRVKSVYCLSSPFIFYGL
AFLLLGMAPFVSNTTGRGWLQNVATGIYAVASASGSLFFAMNFGDEGGAPVKDWIYRACIIQGTQQLYNAALWYW
GTTFTATSSSSGSTVASSYNAFVNSWRMTAITVPVAILMWVVGYLILVGLPSFYRQAPGAVPSFYSALARRKIVV
WTLVTVLIQNFFLSAPYGRNWQFLWASRAAPKWAVALLMVLFFLVIWLGILLVFAKLSKSHSWIVPIFAIGLGAP
RWCQMLWSTSNIGLHLPWAGGAVSSALVSRALWLWLGLLDTIQGVGFGMIFLQTLTRIHVAFTLIAAQVLGSIAT
IIARAAAPNNLGPGDVFPDFSQGANHVLSKAWFWIALLFQLLVCAGYLTFFRKEQLNKP

>g7759: GH31

MARSSLRKVIGVISTGLFLNNAQAQGVTPTPTTATELTATIAGSVVTYSPKFTIPAAADDGANLLPNIHDPLAVD
AQTVCPGYTASNYARNAHGFSASLKLAGEACNVYGTDVDELNLNVQFQNADRLSVQITPTNVDASNSSWYVLPET
LVPRPGLDEDAKSTTIDNDFQITWSNDPTFSFTVLRRSSGDVVFSTAGSKLVYEDQFIEFVTTMPENYNIYGLGE
QIHGFRLGNNYTATIFAADAGDPIDGNIYGSHPFYLDTRYYEVDPETGNKTLVTSNDTSASSTYESVSHGVFLRN

AHAQEVLMRADNITWRTLGGSIDLFFFDGPTQPEVTKQYQVGAIGLPAMQQYFSFGYHQCRWGYQNWTILQEVVD
NFRNYNIPLENIWTDIDYMNRYRDFDMDPNTFSVSEGKQFLDKIHANHQHYIPIVDSAIYIPNPDNATDAYAVYD
RGNSSDVFMKNPDGSEYIGAVWPGYTVYPDWHADESVPWWVNEMVMWYKDINFDGIWIDMSEVSSFCIGSCGTGN
LSLNPAHVPFLLPGEPGNLQLEYPEGFNITNATEASSVAALSSSQAAALSSTAVSTSTESAVTSTSSASTSASYV
RTTPTPGVRNVELPPYVINNINGQLGTHALAPNATHADGVQEYDVHNLFGHQILNATYQALLSVFPGKRPFIIGR
STFAVSGKWAGHWGGDNNSKWPWMGFSIPQALHFGLFGIPMFGVDTCGFNGNSDEELCNRWMQLSAFFPFYRNHN
VIGTISQEPYIWGSVAEATRKAIAIRYALLPYMYTLFHSAHTTGTTVMR**ALAWEFPNDPSLANADR**QFLLGPSIL
VTPVLAQGATSVNGVFPGAGKGEVWYDWYTHSAIHASAGENITIAAPLGHIPVYIRGGSVLPLQQPGYTTFESRQ
NPWALLVALNLDGAATGSLYLDDGESIMQNSTLFVEFTASAGKLSVSATGEFVDANSLANITILGLSRAPVDVAL
DGVLIGSGVSFDGGSGVLSVTGLAGATGGGAWSRDWVLSWS

>g8055

MFVRHRFQELIAFVGLLCVFSSAQDNCTTSIVSTAPPANGTGVQLQQFSYCGGDLNITAYIEDVNYEKIVIVSYS
DRSNKSTPVNSIALDYVSAVTGNERWQLWSAKTPIYIDGITELLKITYKAVNVNKVYTQILNIPVVASGRPAPAP
LAAPVPYATPSGFSDDITSWLAVGALSQIATCKTRMFDNINSVGAANGTVVASTSTANPDYHYNWVRDAALTMDV
VVDLYEAATVPPAVSYYENILFQYSQAR**AGQQQIGGLGEPK**FYLNNTLFTGPWGRPQNDGPAEAAVALIDFASAY
LAKNGSLEKVRKEIYDSTTYPTFAPVKRDLLYVAANWSLESFDLWEEESSYHFFNSLVSHRALTIGSAFAAKLND
SSTASTLSTAADAVVAGMGRFWDENRQLILYEYGPVLKNKTSFKDIAVILGVLHGYADDDIFSYTNDQVLVSAYQ
IATSFLSVYPIAKVTLDSAGGVLGIPIGRYPEDVYNGTGTAPEGGNPWYLATSTLAEFLYKATHSFTNQSSIKVS
NTSLPFWTYFAPSASLAAGTTYPSTSSPFKTAIGALEGWADAFMRRVKFHVPADGRLAEEYHRRDGIATGAEDLT
WSYASVVTAAMARAVVRGDVGYARGLANLGFT

## Glucose metabolism: β-glucan

>g12448: GH3+CBM1

MLALAYLLLSVTSLRLVHADLELKWSYGRSPPVYPSPSGAGTGDWNDAYAKARSALAKMSNAEKANLTIGLTGFT
GCSGTSGGAASIAFPGLCLQDGPSGVRSTDLVNAYPAQLSIGASWNRTLANGVATYMGAEFKRK**GANVALGPTIG**
**PLGR**VALGGRNWEGYGSDPFLSGVLSAEAVLGLQKSVMACVKHLVGNEQETNRNPSIISFQQSVSSNIDDRTMHE
LYLWPFQDAVKAGAASVMCSYQKINGSYGCQNSKVLNGLLKTELGFQGFVVSDWGAQHAGIAGAAAGLDMVMPSA
SFWASNALVTAVQNGSLPQTR**LDDMATR**VLASYYRLGMDSPSYPALGIGIPAVVTAAHPLVEGRDPASKSTLLQG
AVEGHVLVKNVNNTLPLKAPTLLSLFGYDAYAPLVVNPSSSAVDRWTHGVESVTANDVQLLLIAAGLSGTAIGAA
TSGTLTKGGGSGSSYGPYISAPYNAFEQQAHKDGTYLFWDFQNQNPDVSAASNACIVFINEFATESQDRQSLADV
DSDKLVNNVAKKCSNTIVVIHNAGIRLVDAWIDNVNITAVIFAHTPGQDSGRALVEVMYGKQGFSGRLPYTVGRK
QSDYGSLLSPSLPGPILSDTYLYPQSDFTEGLNIDYRDFIARNVSPRYPFGYGLTYTTFSYSNFSITPMTNNLTV
PLPPAPSAQGGNPNLFNTIARVDCTVTNTGTVEGAEVAQLYIGIPNSPPKQLRGFEKKSLQPGERKTFSFPLARR
DLSIWSTTRQEWVLQSGNYQIYVGASVLDIKLQGVLTI

>g15895: GH1

MALVILFSLAAITLATPQSPLEFPNPAGYEFKKYSAPSLDPLWAKIASPIAPPKYTSTVVPTPEPATYTQPNEFH
PLVASHDTNLTNLKLPKNFIWGVASSAYQIEGAAKLEGKGPSIWDALSHNVPNFVADNSTGDNVAEQYMMYKVDI
ARMK**GLGIPAFSPSFAWPR**FFPTGKAKDGANEEAVKHYDDVITELVSAGIKPVITLFHWDTPLALFGEYGAWLSP
KAMDDFVDYAKFVIQRYDSVVSTWYTFNEPQYCNWQFSEYPLDGRFYPIGGQDLSKFVGKEGKLRARFLCGHYTL
LAHARVAKWYHNEFKGKGRITFKNSGNFQEPRTQSAADLRAAQRGFDFSIGWFGGPWTDGDYPTSLRETLGDLLP
TFTTEEKSLIKGSCDFYAIDPYTSFTLYSEPDAENCYTNRTNSGYPECTSSTQTGANGFPMGPSSDNGVSWLKST

PYGIRKFLKKIMVLFPSVPDIVVSEFGFAEPFESRLTKMEDILWDLRRADYYQNYLDAILQSIHYDHINITGAWG
WSVYDNFEWLVGSDVRFGLQYLNYTSLERTPK**ASMFQFLNWFK**QHSA

>g16312: GT48

MSGHPPPPQGGYHDEAYDANGQPYYNDGQGYYDQNQQYEGQPHGAPVAGQDPYYDDQGYYNDNHQGGYAQDGYYD
QNGYQGDEYYDNQYYDQAQGGQPPQGYGYDGGQRRQRRGDSEEDSETFSDFTMRSDVHRAADMDFYGRGDERYNS
YNGEQQGRGFRPPSSQISYGGNRSSGASTPVYGTEFGALPAGQRSREPYPAWTQDAQIPITKEEIEDVFMDLREK
FGFQGDSVRNMYDHFMTLLDSRASRMTPNQALLSLHADYIGGENANYRRWYFAAHLDLDDAVGFANMELGKGNRR
TRKARRAAKKKAAENPADEAKTLEALEGDNSLEAAEYRWKTRMNRMSQHDRVRQIALYLLCWGEANQVRFTPELL
CFIFKCADDWLNSPAAQSGHFVVEEGTYLNTVVTPLYQYMRDQGYEIQDGKYMRRERDHAQIIGYDDINQLFWYP
EGIERIVMEDKTRIVDFPPAERYAKLKEVAWKKVFFKTYKETRSWFHLIVNFNRIWVIHVTAFWFYTAYNSPTLY
TKDYQQERNQKPNPPAQWSAVALGGTLACLIMIIATFCEWMYVPRAWAGAQHLTRRLMFLIGMFALNVGPSVYIF
GFSDQTGKIALALGIVQFFVALATFIFFSIMPLGGLFGSYLTSKKSRQYVASQTFTASWPRLSGNDMWMSYGLWV
LVFAAKMTESYFFLTLSLKDPIRILSVMEMRNCVGDKIVGTILCKYQPIVLLVLMFCTDLILFFLDTYLWYIIWN
SVFSVARSFYLGVSIWTPWRNIFSRLPKRIYSKVLATTDMEIKYKPKVLISQIWNAIVISMYREHLLAIDHVQKL
LYHQVPSEQEGKRTLRAPTFFVSQEDHSFKTEFFPSQSEAERRISFFAQSLSTPIPEPLPVDNMPTFTVMIPHYG
EKILLSLREIIREDEPYSRVTLLEYLKQLHPHEWDCFVKDTKILADETSQFNGENEKNEKDTARSKIDDLPFYCI
GFKSAAPEYTLRTRIWASLRSQTLYRTISGFMNYSRAIKLLYRVENPEVVQMFGGNSDKLERELERMARRKYKIV
VSMQRYAKFTKEERENTEFLLRAYPDLQIAYLDEEPPVEEGDEPRLYSALIDGHSEIMENGMRRPKFR**IQLSGNP
ILGDGK**SDNQNHAIIFYRGEYIQLIDANQDNYLEECLKIRSVLAEFEEMTVENVSPYTPGLPPPSTTPVAILGAR
EYIFSENIGILGDVAAGKEQTFGTLFARTLAQIGGKLHYGHPDFLNGIFMTTRGGVSKAQKGLHLNEDIYAGMNA
LLRGGRIKHCEYYQCGKGRDLGFGSILNFTTKIGTGMGEQMLSREYYYLGTQLPLDRFLSFFYAHPGFHINNLFI
ILSVQLFMVVLINLGALKHETITCTFNKNLPITDPLKPTGCANLVPIENWVARCIVSIFIVFFISFIPLVVQELT
ERGFWRAATRLAKHFSSLSPMFEVFVCQIYANSISANLSFGGARYIGTGRGFATARIPFGILYSRFAGPSIYVGA
RLLMMLLFATMTAWGAWLIYFWVSLLALCICPFLFNPHQFAWNDFFIDYREYLRWLSRGNTRSHSASWIGFCRLT
RTKLTGYKRKALGDPSSKLSGDIPRARFTNIFFSEIISPLILVAVTLIPYLFINSQRGVTADLNPDKTVEATNSL
IRVGLVALAPIGVNAGVLAAFFGMACCMGPLLSMCCKKFGAVLAAIAHAIAVIMLFAFFEVMFFLEGFSFAKALL
GMITVLAIQRWIYKLIIGLALTREFKTDTANVAWWTGKWYSMGWHSVSQPGREFLCKITELGYFAGDFILGHILL
FIMLPALLVPMIDTVHSVMLFWLRPSRQIRPPIYSLKQSKLRKRRVIRFAILYFVMLVLFIALIVGPLVAGKYIT
GFTLPLQLAQPTGLNRNDTLSSETGTAVNGGAAATDAASSTVAARRLLARHY

>g16315: CBM43+GH72

MRGLSAVAGVAALFARSVVADLDPIVVKGAKFFYKTNGTQFFIQGVAYQQDYSTNGSSSTTASSAYTDPLANAAR
CRVDIPLMKQLNMNTIRVYAVDPTQDHTACMQLLQDNGIYVVADLGQPGLSINR**DSPAWNTQLYAR**YTSVVDMFA
PYSNVIGFFAGNEVSNNKTNTNASAFVKAAVRDTKAYIKAKNYRQMYVGYATNDDAEIRANLETYFNCGDTSEAI
DFWGYNIYSWCGDSSFTESGYDQRVAEFKNYSVPTFFAEYGCNTVQPRKFSEVKAIYSSQMTGTFSGGIVYMYFQ
EANDYGLVQVSGSTVSQLADFTYLSSQMATIAPTGVQMASYTPTNSPQACPAVQTGVWEAKASPLPPVANAQLCS
CMVNSLQCVVKSSQAENTYGTLFNQVCGYGSSCAGIAAIASNATYGAYAACNSTQQLSFAFNQYYLSQNKASDAC
NFGGSATTKAASTPSGCASLISQAGSAGTGTVSSGASSTATKKSAAGVTSVPSFNINLFGLGIYVSMAVVVGAGM
ILL

>g1789: GH17

MHAAQSFVALATLASVASAQVMGFNSGATLDTYKVKTQSDYEAEFTTAQGLVGAPGKFNSVRLYTMIQGGTDTDP
TSAFQAAIKTNTTMLLGIWCSGTTTIEKELKALSTAITTYGAKFTDLVVGISVGSEDLYRTSVTGIINK**SGIGNS**

**PDAIVNFIK**DTRKAIANTPLSGTPVGHVDTWTDWTNSSNKAVIDAVDFIGNDLYPYYEDTKDNSANNAVELFNEA
YNATLAAAGGKPVWITETGWPTSGPLFGKATASNSDAQKYWQTVGCQLFGKTNVWWYNLRDSNPANEAKFAITSD
LSTTPSFNLTCPAVVKTTKGGDGISSKSNSTSTATFPGSTGTSSSSSGNVTTGSSAGARTSGAAGSGTSPSSPVV
TGVASSAGAMMGLTMCILLGSISLLL

>g21360: GH72

MKASSAFVATCALFSSVIAGSVSRR**ANTISNSNTPPVSVKGNAFFTSK**GRFYIRGVDYQPGGSSLLKDPIADLDG
CKRDVAKFKELAINTIRVYSVDNSADHDACMKLLADAGIYLALDVNTPYYSLNRKDNASIAMSYNAVYLQSIFAT
IEAFAKYDNTLLFYSANEVINDDSTTFAAPYIKAVTRDMKAYMKARSLR**AVPVGYSAADIESNR**YQTATYLNCGP
DAARSDFFAFNDYSWCDPSSYTISGWDKKVATYSNYSLPLFLSEYGCNKNKREFEEVKALYGSNMTPVYSGGLVY
EYSQEEADYGLVDISGNTVTERPDFTALK**SAFAGTANPTGDGGYK**SSGSASPCPAK**SDIWEVEDPTILPNIPSGA
TKYMTSGAGAGPGNK**GSTGSQTAGGASTGWSTTSSSGSTTSGSASSATSSKAAAGNLQVPQLSMAPFVVAAVAGL
SGLIGGAGFLL

>g21591

MLALHRTLLGLLAFASTSLAVNTVTIQGQDFVDTVTKNRVMIIGVDYQPGGQGGYDPNVRADALSNGTVCLRDAA
LLQKLGVNTIRVYNVDPNANHDLCASIFNTAGIYMIIDVNSPQQSINRADPSSSYTVDYLTR**IFAVVEAFK**GYPN
TMAFFSANEVMNDIDTGKSNPPYIRAVQRDLRQYIAKNSQRTIAVGYSAADVRPILQDTWAYLQCNANSTDDWSR
SEFFGLNSYSWCGADATYQTAGYDQLVSMFQNSSVPVFFSEYGCNKPAGLARPFNEVQALYGPQMTSLSGGLVYE
YSQEESDYGLVVINANGSITLRGDFDNLQNQYNKLNVTLLQSTAAGNTQITPPQCSASLITNSGFSKDFTVPAQP
SGAAALINSGISSPNQGKLISTGDLNVKQQIYSSSGKLITGVAVKAVSGANTPGGENTSGSSATSTSTSSGTASP
SASKKAAAASLQVTEGVMMRGLILASAIALGSGLIWRP

>g6964: GH3+CBM1

MKLSLVAAASLLVVSATATSPKLRSRQYSNSTTSSNPGAQFGQTSPPYYPSPWMDGSGGWETAYQKAQAFVKQLT
LLEKVNLTTGVGWEGEACVGNVGEIPR**LNFPALCMQDSPLGVRSADYVSAFPAGGTVASSWDRQVWYQR**GHDMGS
EHR**DKGVDVQLGPVVGPLGR**APEGGR**NWEGFSPDPVLSGIAVAQTIK**GIQDAGVIACTK**HFIGNEQEHFR**QSPEA
ASFGVNISESISANIDDTTLHELYLWPFADAVRAGTGSIMCSYNQVNNSYACQNSYLLNNILKGELGFQGFVMSD
WQAQHGGVSTSLAGLDMSMPGDTVFNSGISFWGANLTLAVLNGTVPEWRIDDMATR**IMAAYYLVGR**DTK**KVPVNF
ASWTK**DTFAYRHPAVNSR**YELVNQHVDVR**AEHFRNIRDHAAKSTVLLK**NSGVLPLTGK**EKFTGVFGEDADTSAWG
PNGTPDRGSDNGTLAMGWGSGTADFPYLVSPLTAIQNELVKNNALVQSVTDNWAYAQIASLASQVSTAIVFVNAD
SGEGYIAVDGNIGDRNNLTVWRNGDTVIQNVTAKCNNTIVVIHSVGPVIVTDWYNNPNVTAILYAGLPGEQSGNS
LTDILYGRYNPGGK**LPFTLGAK**REDFGTDLLYTPNNGGNAPQDQFTEGVFIDYRHFDKAGIKPIYEFGFGLSYTT
FAYSGLQVQSHSVNTYTPTTGSTSAAPVLGTFSNNTADYLFPSNFTRVGLYIYPYLNSSNPATASQDKDFGKDNL
PAGSRDGSPQPRIAAGGGPGGNPQLYDVLFTVSATIQNTGQVEGDEVVQVYVSLGGPKDPVRVLRQFDR**LTIAPG
ATATFQADLTR**RDVSNWDTVAQNWVISNYTK**TVFVGSSSRTLPLSQTLTFGSY**

>g7121: GH17

MFAKLLALAALPAASNAFGVLK**GFNYGSTDASGVVK**DQAR**FEQEFSTAQNLVGTSGFNSAR**LYTTIQGGTTNSPI
SAIPAAISTQTSLLLGIWTSAGQAIVDNEIAALKAAISQYGTAFTDLIVAVSVGSEDLYR**NSGYPGASDPGPGAN
PDVLANYIGQVKAAIAGTSAEGR**LVGHVDTWTAFVNSSNNALISAADFLGVDAYPYYESANGNDISNAANLFASA
YSQVVAVAQGKPVWVTEAGWPVSGPTVAQAVASPENARSFWTSVGCNQLFDKINVWWFQLDDYPTSPNPAFGVIG
TAFSTTPLFDLSCPATTKSRRRSNRAA

## Glycoprotein and mannose metabolism

>g12843: GT66

MDALFQGDAAKNTRTLLRAIILLTIAGAAISSRLFSVIRFESIIHEFDPWFNFRATKYLVQHGFEPFWNWFDDRT
WHPLGRVTGGTLYPGLMVTSGVIYHFLRLISLPVDIRNICVLLAPAFSGLTAYATYLLTSEMSTSPSAGLLAAAF
MGITPGYISRSVAGSYDNEAIAIFLLVYTFYLWIKAVKEGSVMWGALAALFYGYMVSAWGGYVFITNLLPLHAFV
LICMGRYSARLYVSYTTWYAIGTLASMQIPFVGFLPIRSSEHMSALGVFGLLQIVGFVEYVRLQLPSKQFQTLLR
SLVLLIFLVSFGGLVLLTVSGVIAPWTGRFYSLWDTGYAKIHIPIIASVSEHQPTAWPAFFFDLNLLIWLFPAGV
YLCFRTLKDEHVFIVIYAVLSSYFAGVMVRLMLTLTPVVCVAAAMALSQILDIYLLAESPSEELQTLSSAEAAK**A**
**AAGTSLLSDGLR**STTKPIVGIYSYMSKATVVVCSTIYLLIFVAHCTWVTSNAYSSPSVVLASKMPDGSQHIIDDY
REAYYWLRQNTPQNAKVMAWWDYGYQIGGMADRPTLVDNNTWNNTHIATVGKAMSSREEVSYPIMRQHEVDYVLV
VFGGLIGYSGDDINKFLWMVRIAEGIWPDEVKERDFFTPRGEYRVDDEATPTMKNSLMYKMSYYNFNALFPAGQA
QDRVRGSKLPAQGPELSTIEEAFTSENWIIRIYKVKDLDNFGRDHSNAVAFEKGHKKKKAAKRRGPRSLRLE

>g18706: GH31

MLQMGTQSKGWSRTLSLLCLVGLFTPVFTVKHENFKTCDQSGFCKRNRQYADAAGTAAFTSPYELESSSISFQNG
QLKAAVIKTVGKSGEKVRLPVTISFLESGSARVTLDEEKRVKGDIELRHNSKARKERYNEAGQWAIVGGLAPSAG
AALNNAAEKGTTIVKYGPSGTFEALVRHAPFSIDFKRDGETQIQFNEGGLLNMEHWRPKIEKKVEEPKEGEAGPA
APAPEDPNAEDEGTWWEESFGGNTDTKPRGPESVGLDISFPGFEHVYGIPEHASSMALKQTRGGDGAYSEPYRLY
NADVFEYELDSPMTLYGAIPFMQAHRKGSSVGVFWLNGAETWVDVVKSKTNANPLSLGIKGSTTTQTHWYSESGQ
LDVFVFLGPTPKDVIKSYGELTGYTQLPQEFAIAYHQCRWNYVTDDDVIDVDKKFDKFKIPYDVIWLDIEYTDGK
KYFTWDPLTFDDPENMGKQLAKRERKLVTIIDPHIKNTDSYHVVDQLKSKGLAVKNKDGDIYEGWCWPGSSHWVD
CFNPAAIAWWKSLFAYDKFRGTLPNTFIWNDMNEPSVFNGPETTMPKDNLHHDNWEHRDVHNINGMTFQNATYHA
MLARNKAEEKSPRRPFVLTRSFYSGSQRVGAMWTGDNLAEWSHLAVSLPMILNQGISGFPFAGADVGGFFGNPSK
ELLTRWYQAGAFYPFFRGHAHIDTRRREPYLAGEPYTSIITKALQLRYALLPSWYTAFHEASTTGAPIVRPNFYV
NPADEAGFTLDDQLYLADTGLLFKPVVTEGAESVDIYLGDDAPYYDYFDYTIVKGKGSHTLKAPLDKIPLLMRAG
HIFPRRDRLRRSSGLMKLDPYTLVLVLGPDGKAEGELYVDDGESFDYEQGAYIHRKFIYENGSLRSEELGKKGKL
TDKYAKKMEKVRVERVVIVGAPSAWKGKKQVLVSEEGEKDSKGGKKVK**IDFTDATAGK**AAFAVVRDPKVAVGKGW
KIDFGA

> g2667: GH63

MLTPKFASMISGLLLVSTAIATPLSSPGNHQKRAIPRSSVPPTMAPYDRADYVYDPEKEESGFDRRTARTFDSDA
GASHTTKRSPTDLERRYDGWEATPICNKESFGGKADYNQVNELMDTLLMQNGKPEVGAGPKKCAMAGSTLGLSLS
LTFGLRASRSFISRTNTTIANAIMRSRLQSPSPWPSLLSSLLLLLIAPITSASTSQPTTIADIERASNQSLLWGP
YRPNLYFGVRPR**IPESLLIGLLWAK**VEDYQSVQHNFRHTCEQHEGMAGYGWERYDPRHGGVQTIHDAGNQIDITT
SFFKETGEGSGDRGGNWGVRIKGVPRADAQEDLKSTVVFYASTEGQGLNNRLEVKNVDELQGGGFDGDVVLKGEN
LGLGEYKIVVKGDEGTENKHPSVTHPSGSEKDLGKTLVKSSTVPEDAIWQTKPILFAMLKEQIDEYVEKYTKENP
PPPFQLYTIKSEAGSGNVHFVQKVFEGAFEFDVLFSSSAAESELASSDLSKGISEIVKTFDTRFDSIFKPAAPFN
SGKYLDFSKSLFSNLLGGIGYFHGNSRVDRSYAPEYEEDNEGFWEEAAEARARADVK**LEGPNELFTSIPSRPFFP**
**R**GFLWDEGFHLMPVVDWDIDLTLQIVK**SWFALIDEDGWIGR**EQILGAEARSKVPEEFQVQYPHYANPPTLFFILS
AFVDKLTETAPSSKDAEYSPQLLDKEVATSYLKELYPLLKRHYNWFRKTQQGDISSYDRKAVNTKEGYRWRGRTP
RHILTSGLDDYPRAQPPHPGELHVDAISWVGLMATSLQKIGLFLNEKEDVEKYTKQLTGIRSNIADLHWSEKDGV
FCDATIDEFEENSLVCHKGYISLFPFMLGLLDPADDGNKIAKILATIGNKEELWSEHGIRSLSIADEAYGTDENY

WRSPVWINMNYLIVSR**LVALAQDPTAGSDNQK**TATKLYTDLRINLVETVYKSWKETGFAWEQYNPETGAGQRTQH
FTGWTSMIVKILGMPDLSKGSAKVRDEL

>g3844

MGGSHDTPTSTKYPTLVQKPVGKQIHNLYLDRLQQFTDNGQYRKQGLLDKIIEARASGDQWVRLEVYSPPDLSRP
TFKEATSHKFRDTHVGESFGPSWATHWFKIHLTIPDDLAKKEHLEFVWDANNEGMIWTEKGDVVHGLTGGGDR**TQ**
**WILPESWR**DGKEHIFYVEMACNGMFGNAPGGDSIQPPPPDKYFQLHTAEITAVNLDARQLYIDFWIIGDGAREFP
GDGWESHKALQVCNAIMDCFIAGQGTKECIKECRKIAREYIGNVDTPKIYDGDLPSLVTAVGHCHIDTCWLWPWA
ETKRKVARSWSNQCDLLDRYPELRFCASQAQQYKWLEMLYPSLFDRVKEHVKKGNFQPIGGSWVEHDTNMPSGES
LVRQFVYGQRFFESHFGQRCTTFWLPDTFGYSSQLPQLCRLAGMSRFFTQKLSWNNINNFPHTTFNWVALDGSQV
LCHMAPSETYTAEAHFGDVKRSITQHK**SLDQDETSLLVFGK**GDGGGGPQWEHIEKLRRARGISDTVGLLPRVK**LG**
**DSVDDFFAK**LEKKAETGTDFVTWYGELYFELHRGTYTTQANNKRNNRKSEFMLRDIEFLATMATIKDDVDGKKST
YKYPKKEIDFMWEAVLLCQFHDCLPGSSIEMCYDDSDKLYAEVFETGTKVLTDALSELGFDDDKKTSSVGDLVAL
NTLPWARSEMTRLPIKSEAPKYAAIDSTHTGLGVVRALTAASSAPVSIRETEK***GSFELSNSAFDVK***MSDGVITSL
FDKRANREVIAKGGKANQLVIFDDKPLYWQAWDVEVFHLQSRQELSSSTSKIAEQGPHRVSVVTETKISAESWVK
TTISLNAATDDNYASIDVEAEVEWHETMKFLKVEFPVDVSNTEASYETQFGIVRRPTHYNTSWDMAKFEVCCHKW
ADLSESNYGVSILNDSKYGFATCGNLMRLSLLRAPKAPDGHADMGRHQIRYSIFPHNGPLDYKTIRAGYSFNNPM
KLHHHPKPASISSLLSSFNIDGSKSLIIDTVKRGDDDVDVSRGELPAKKGKSVILRIFDALGGKSK**GILTWGDVP**
**VK**AVFK**TNLLEDAGDELVVLK**SGKGVEIELRAFEVATFRLELQ

>g9548: GT24

MRVPSWLLPAEILLLVGSLGLVPLTGAAPSINVGLHASFNSAPYLVELLETAAEEKPDVYYKILDRISDGYFSDA
STDKELYEKFLRLLKYDNLITDSESLSSFELALSIHNAAPRIEAHYQYYKTTIEPLLEKSKGKDCETWLAFTGKQ
FCSPQFEKADATIK**GINIEGGVPILPFDR**IFGSPTTIPAVIYADISSPVFKKFHSLISEAAKNGRVSYRVRHKPS
KSERKPLEMSGFGTELALKRTDYIVIDDRKAEEGKEADKESSKPADIDLVDEEVADLKPLTSSEVTELGLK**ASTY**
**VLGSEDPLETLVK**LTQDFPKYSSVIAGVNSSEAFLEEHRENRALLLPAGFNVIWINGVQFDSRKVDAFSLLDHMR
RERALLGSFQEMELTSEEAIKLLTHPSIAEAQSDVDVQRYDWRDETEGGNVIIWMNDIEKDKRYAAWPSEIYGLL
QRGYPGQLPTVRKDIHNAIIPIDFSEPTSLHRATETVQDFVKRKIPIR**FGLVPITSTAGAAK**QAQVVYHILDTYG
LGALMQYLESSLSAKKTAAPHESTFKAVIEKRKPKAGREAVALQAVLQSEESAIQVGGAKKYLTRLGATGSTPPV
FMNGVPIKNDDEWLQAMVQR**VSQDLQWLQR**GVFEESITQDMWIPSNWLNDSSIRRNALVIPENHKDIK**LLNLLNV**
**LK**WEDEQTMKIMPTIPADEDSDKAKWAQLVLVGDFDTQVGLQMILDAIKFRRENPNIELVLVAQSGSPMIKSPRI
VQIWPKETKWTMETIQAVYRDIKALLAAPYDHAAHDNSQFWPVETEQLAQAFGFDQGQNGLIINGRKIGPISAES
AFTKDDFAALYKFEFKKRIAPASEAIAELEYKDKIKTVSDAAKLCSLLAVSTVSDMPEGIFELPPPLRTRAYYEW
SDSETAVTVGDNTTALINIVAVLDPAAEPAQRWSPILRVLSKLEGVSLKLFLNPKENLHELPVKRFYRYALNEKP
SFDDDGAVTAPGVKFEGIPKDTLLTVAMDVPPSWLVAQK**ESLYDLDNVKLSSLPLGDNVDATYELENILIEGHTR**
DSKGGDQPPRGAQLVLSTAKDPHFADTIIMANLGYFQFKANPGYYNITLKPGLSSKIFKLDSAGALGYEATAGDE
ISEVCLLSFQGLTLYPRFSR**NPGMEDEDVLGSTKSSTASELASK**GADLVDGFLNKAGIKKTKGVQSAQADINIFS
VASGHLYERMLNIMMVSVMRHTQHTVKFWFIEQFLSSSFKSFLPTLAKEYGFKYEMVTYKWPHWLRAQKEKQREI
WGYKILFLDVLFPLDLDK**VIFVDADQIVR**TDMYELVTHDLKGAPYGFTPMCDSRVEMEGFRFWKQGYWKNFLRGL
PYHISALYVVDLKRFRQIAAGDRLRQQYHQLSADPASLSNLDQDLPNNMQMMLPIHSLPQEWLWCETWCSDESLK
DAKTIDLCNNPQTKEPKLDRARRQVPEWTEYDDEIAALARRTKQSEKVPTGDEQAGTEGRPKESVVESESAESTH
VRDEL

## N-acetylglucosamine metabolism

>g21234: CBM18+GH18

MKLSTTLSLLTAGLAAASSCTKRTTTGRNVMYYDQYHTNLTTLTPEIASGITHVIIAFIPSTNFTVANTSAFVPF
ESVSKVRTRFGNSTKVMIAIGGWGDTAGFSIGAKTNESRALFAKNVKKMLLETGANGVDLDWEYPAGNGADYKTN
PNSNKTDEIITYPLLVQAISEAIGSSYLLSAAVPGLAR**DMIAYTTATGPAIFK**YLDFVNLMSYDLMNRRDTVMKH
HTSIADSITAVDLYTSIGLAPSKINLGIAFYAKWFAAAPNGTCDAASPLGCKTALLEDPVTGDDLGLAGAVTFEA
SNYAIVDETKLANSTDGSCGANVGPFGTRCIPGNLDWISRESNPVQSSGITDLGRTIALGRQLVFQSDLNKKWPG
ASCVTLVPPESNSSSTLAASQEILPGDTTRMEFQEAAAAFRYRS

>g2198: ViCHS3b

MAHQGYGGGGYNDPSQPPGSQYHAPGSRRGSDEEHEVAQSLLHADPTGTREGPFGNQYASEHTDRMRTPEVRPTS
TYSLSETYADNTGYGPGYNQGYAEDQQAGYDMPPRIASPYSRSETSSTEAWRQRQQPGGGAAAGGGLKRNATRKI
KLAQGAVLSADYPVPSAIQNAIQAKYRNDLEAGSEEFTHMRYTAATCDPNDFTLKNGYNLRPAMYNRHTELLIAI
TAYNEDKVLTARTLHGVMQNIREIVNLKKSEFWNKGGPAWQKIVVCLVFDGIDPCDKGTLDLLATVGVYQDGLMK
KDIDGKETTAHIFEYTTQLSVTANQQLIRPLDDGATTLPPVQMMFCLKQKNTKKINSHRWLFTAFGRILNPEVCI
LLDAGTKPGPK**SLLALWEGFYNDK**NLGGACGEIHAMLGRGWK**NLLNPLVAGQNFEYK**ISNILDKPLESSFGYVSV
LPGAFSAYRYRAIMGRPLEQYFHGDHTLAKILGPKGIDGMGIFKKNMFLAEDRILCFELVAKAGSKWHLTYVKAS
KGETDVPESAPEFISQRRRWLNGSFAASIYSLMHFGRFFKSGHNPIRKFFFVLQMFYNVAMLVLSWFMLGSFWLT
TSVIMDLVGGTVDEIKNPTATTASKGWPFGIKYSPHVNAVLKYIYLGFVILQFILALGNRPKGSRISYIVSFCVF
AIIQLYLIVLSFYLLGKALSGNTVKDNFNDLEKFFSPDGVGVILIAVIATFGLYYIASFLYFDPWHMFTSFPQYL
LLMPSFTNILNVYAFSNWHDVSWGTKGSDKSEALPSAKTEKSSDGKHTVIEEVDLAQADIDSQFEATVKR**ALSPF**
**VATPEDNTK**TTDDGYKSFRTKLVSTWIFSNIIIVIVITSETFDFIFPASSASTKRTATFFTALLWVTAGLSVIRF
SGCIFFLFKTGALRITRKR

## Trehalose metabolism

>g17565: GT20

MSLEPLQMEGRLLLVSNRLPITIKRNDEGKYDFNMSSGGLVSGLSGLKNDVTFEWYGWPGLEIPDDEVGDLKTKL
KEEYNAVPIMLDDELADRHYNGFSNSILWPLFHYHPGEITFDESAWEAYTEANRLFAKAIAKDVQDNDLVWVHDY
HLMLLPAMLREELGDTKKNVKIGFFLHTPFPSSEIYRILPVRNEILLGVLHCDLIGFHTYDYARHFLSSCSRILG
LPTTPNGVEYKNK**VVTVGAFPIGIDPEK**FAEGLKKPKVIERIETLKRKFQGVKLIVGVDRLDYIKGVPQKLHALE
VFLTEHPDWIGRVVLVQVAVPSRGDVEEYQNLRSVVNELVGRINGKFGTIEFMPIHFMHKSVSFDELVALYAVSD
VCLVSSTRDGMNLVSYEYIATQAERHGVMVLSEFTGAAQSLNGSLIVNPWNTEEMAEALHEAVTMGDEQRKINYD
KLARYVNKYTSSWWGQTFVTEMVRMTEQTEKKLSIRSGSKVAFADKESAASTEEKANEPTGGLTVTDGHLVSSSS
ASDSPTSGGEANEKPVVTSPESPVKEAPETEGNRAPAESATPEKSHTL

>g315: GT20

MTSPPKGKQGDAQETGLLSRTRTNSDDSFHAHLVSNAVPVTPGVHTAGHSAYFEQKREEGEPDQEEFHDDDASPG
PNWNANNYHVPKTDDGPPVSPGLAATDAKTAQEAIRKLTMAASGDSGKKELSDVDPRAAHPQLGLSGHIISATFV
VPYNISFAPGKDWELKPRSGTSALFDSFSYLASSSSPWNHTLLGWTGEIKKNPAAFPSNPALAAMENLNSTKKTS
IPVEGKMKLVDPTQSTSMKVSRKDRQRLETQLERDHGGKIVPVWLVDDVSEDDDIYTIENQSRWRSFAERELYTL
FHYKQNEPSDGRGVRTAWADYYKLNKLFADRIIEVYKPGDIIMIHDYNLMLLPSLIRQRLPKAYIGFFLHIPFPS
SEYYRCLSRRKEILEGVLGANLIGFQSFTYSRHFSSCCTRILGFDSSSSGVDAYGVHVAVDEFPIGINAFSTKKA

AYNDPMVEEKMAGILQLYAGKRIIIGRDRLDTVRGVSQK**LEAFENFLER**YEEWRDKVVLIQITSPNATNTVDDGG
ESKFMDKISDRVSKINGKYGSLSFSPVKHFPQYLSKEEYFALLRVANVALITSVRDGMNTTSMEYVVCQENNFGP
LILSEFSGTSGSLKAAIQVNPWDLGGVADALNSALHMSNEERKEKHAKLYRHVVNNNVQNWTNKLFKRFLINLES
FDQSFSTPALDRVKLLTAYRTAKKRLFMFDYDGTLTPIVKNPESAIPSDRVLRTLK**TLAADPANTVWIVSGR**DQA
FLDHYMGHISALGLSAEHGCFMRQPESDDWENIAAHMDMSWQQEVKNTFNTYTDKTPGSHVEVKKVALTWHYRNS
HPELGLEMSRKCQRELETTVARNHDVEVMTGKANLEVRPKFVNKGEIARRLVKAYGSGPGDAPEFVFCLGDDSTD
EDMFRALKQSELPSGNVFSVTVGASSKQTLASWHLLEPHDVISCISLLNGSIDNENVGAVSVVDGTIPEMTEARI

## Galactose metabolism

>g20534: CBM87+CE18

MFALRRLGSALVASLSLASLVSAANTVNSTILVFARDTASGYSGTSGLAGYGIPYQLVVVPQAGITLPVLNSTAT
AGNFGGIIILSDVAYSYDSGWASAITAAQWAQLFAYQTSFGVRMVRLDVYPGSNFGTTTAIAGEGCCDAGVEQLV
SISSNTAFPKAGMNTGAGVTTQGLWHYPAIINNASIATEIAQFAPAGDFTTTTTAAVINNIGGRQQMAFFMGFAT
DWSSTSNFLQHAYIHWMTRGLFVGRRRIYFNTQIDDMHLVTDIYQPAGSLYRVVPNDMVTHVSWVNGLNSR**LPAG**
**SSYK**VEIGHNGNGAIENALTIDPTSCTPNSAIEYAEQIDTPLEFQKPLGTGTNIWPATPTLYPAGWGTACLNKDP
LAAWFRVAANRNAFFHISHTFTHEGLNNATNSDANKEIAFNRAWFYYMGLDSAATFSTTGIIPPAITGLHNGDVI
RAWIANSIMHVVGDNTRPPLLNTVNEFWPLTSTVAANGYAGLTILPRWATTIFFNCDTAACTTAEWVNTSGGKGD
FAALLANAKATNTRHLFGLHQDGFMFHQANLRADSAIPSYTVGSQSVQSLLQIWVETITQEMSRLTTWPLISLRQ
DDMATQFRNRQTRDGCSPNMVWNYSADNKKIVGATVTANRNTCSVPIFATFPSALTTSPGTNDGVGSDSLQYPIT
LSGAAKTYTFSTAISV

## Effector candidate

>g18338: ViEcp6 LysM

MLFAKSSVAMVSLFTLLVAAAPAPELLSARELCNGTITLDERVQK**YTIASGDSLGAIATK**FNRGICDIATANKIT
NINFVTAGQVLTIPAQVCIPDNASCQPKTPEATATSILGGPGFYIVVSGDTLTAIAKNFQITLQSLIAANPAITN
PDLILVGQVIIIPVLPGSSCTISPYVIKSGDIFFDLAAK**FGTTAGQLLSLNTGTDPTK**LAIGQTVTIASGCKNAT
ATTGENKYWKGQWGGPGGKWEGKWGN

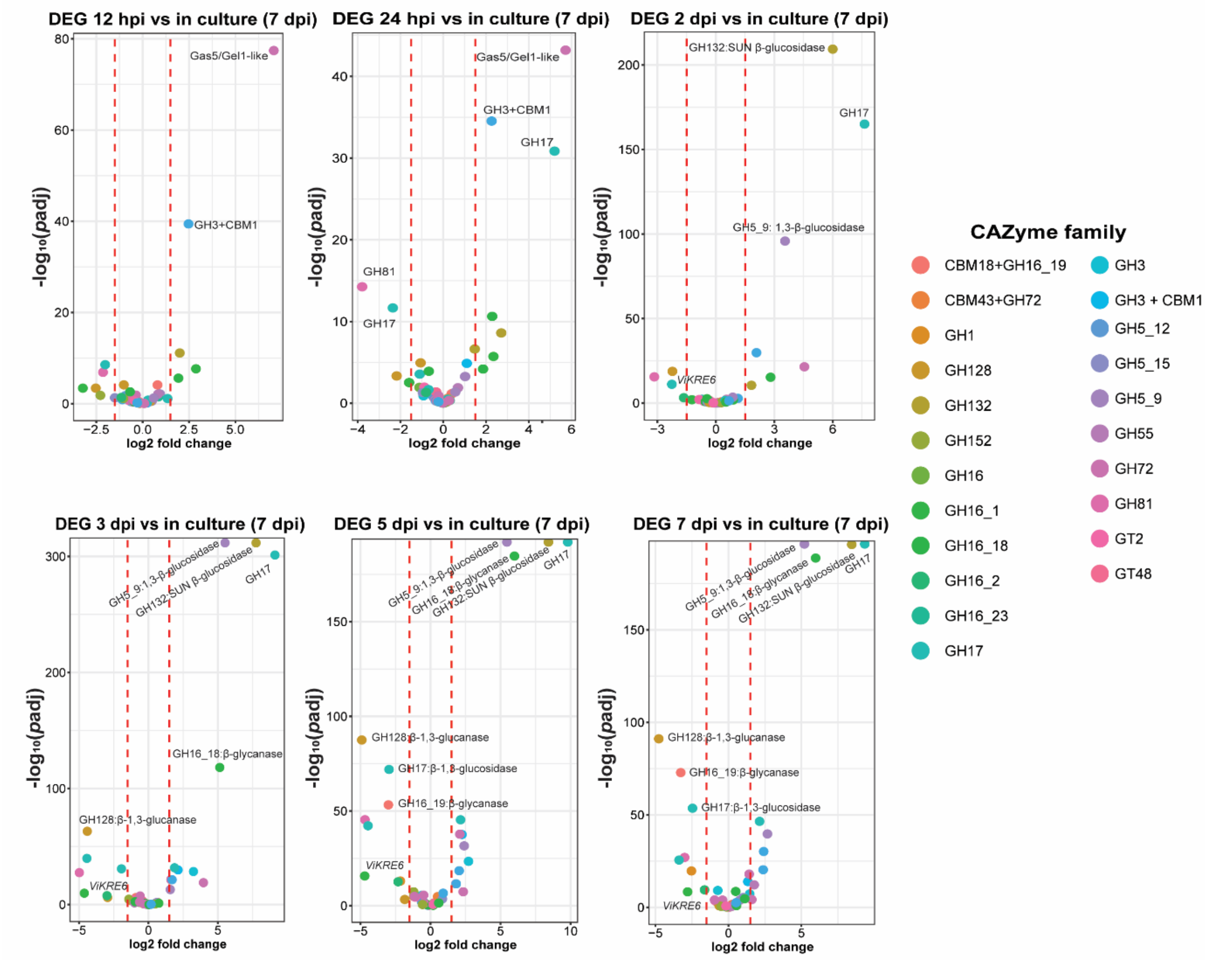


**Figure S3. Volcano plots illustrating genes of *Venturia inaequalis* that are both putatively associated with β-glucan metabolism and are up- or down-regulated during one or more *in planta* time points compared with growth in culture on the surface of cellophane membranes (CMs) overlaying potato dextrose agar.** *In planta* time points were at 12 and 24 hours post-inoculation (hpi), as well as 2, 3, 5 and 7 days post-inoculation (dpi), while growth in culture was at 7 dpi. Dashed red lines indicate the 1.5 $log_2$ fold change used in this study to identify significant (*p*-adjusted, *p*adj) differentially expressed genes (DEGs). Each dot represents one gene, coloured by carbohydrate-active enzyme (CAZyme) classification. Only genes with a minimum expression of 10 transcripts per million (TPM) at one or more *in planta* time point are shown. CBM, carbohydrate-binding module; GH, glycoside hydrolase; GT, glycosyl transferase.

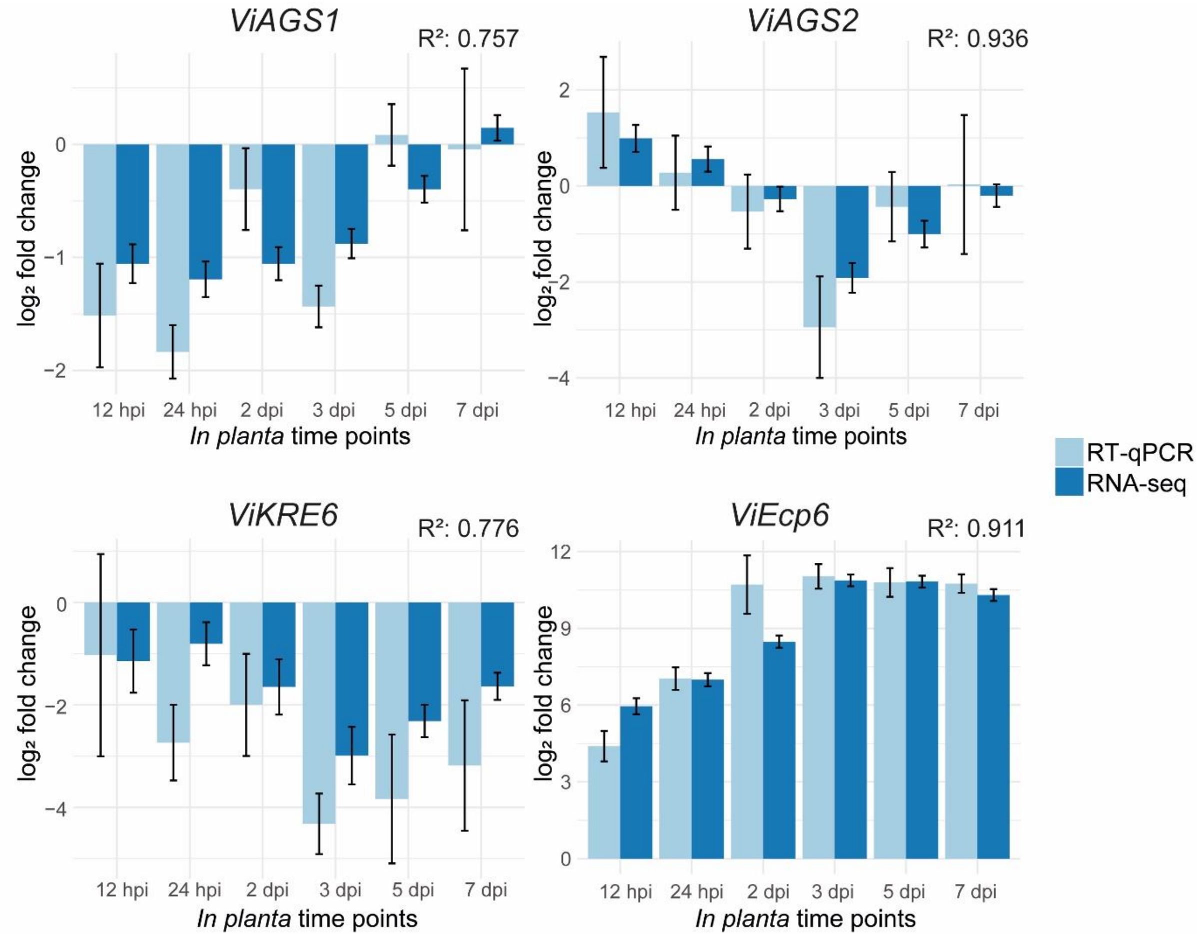


**Figure S4. Validation of *Venturia inaequalis* gene expression changes observed during host colonization by RNA-seq using real-time quantitative PCR (RT-qPCR).** *In planta* gene expression levels were compared to gene expression levels *in culture* on the surface of cellophane membranes (CMs) overlaying potato dextrose agar at 7 days post-inoculation (dpi). *In planta* time points were at 12 and 24 hours post-inoculation (hpi), as well as 2, 3, 5 and 7 dpi. The genes selected for validation were *ViAGS1* (*g748*), *ViAGS2* (*g4310*), *ViKRE6* (*g7748*) and *ViEcp6* (*g18338*). Relative expression of target genes was calculated with the $2^{-\Delta\Delta Ct}$ method (Livak and Schmittgen *et al*., 2001) using the geometric mean of two housekeeping genes, *β-Tubulin* (*g10951*) and *60 S ribosomal protein* (*g3362*), as reference. For all genes, a very good Pearson correlation ($R^2 \geq 0.7$) among RT-qPCR and RNA-seq gene expression levels was observed. Error bars represent standard deviation across three biological replicates.

**Supplementary file 5**: **Raw real-time quantitative PCR data and primer sequences for validation of *Venturia inaequalis* RNA-seq gene expression data.**

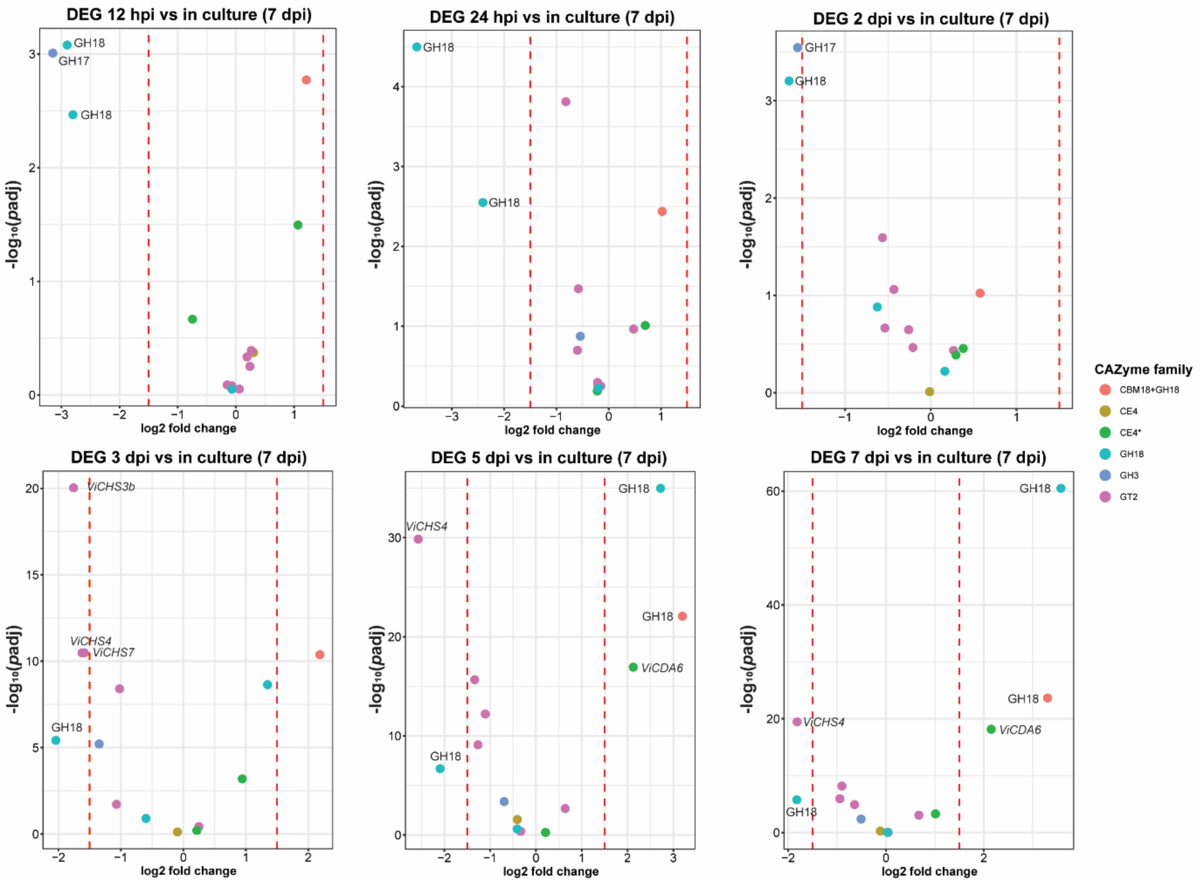


**Figure S5. Volcano plots illustrating genes of *Venturia inaequalis* that are both putatively associated with chitin (N-acetylglucosamine) metabolism and are up- or down-regulated during one or more *in planta* time points compared with growth in culture on the surface of cellophane membranes CMs overlaying potato dextrose agar.** *In planta* time points were at 12 and 24 hours post-inoculation (hpi), as well as 2, 3, 5 and 7 days post-inoculation (dpi), while growth in culture was at 7 dpi. Dashed red lines indicate the 1.5 $\log_2$ fold change used in this study to identify significant (*p*-adjusted, *p*adj) differentially expressed genes (DEGs). Each dot represents one gene, coloured by carbohydrate-active enzyme (CAZyme) classification. Only genes with a minimum expression of 10 transcripts per million (TPM) at one or more *in planta* time point are shown. CBM, carbohydrate-binding module; CE, carbohydrate esterase; GH, glycoside hydrolase; GT, glycosyl transferase. Asterisk (*) indicates CE family 4 (CE4) proteins were only predicted using a protein family (pfam) domain search.

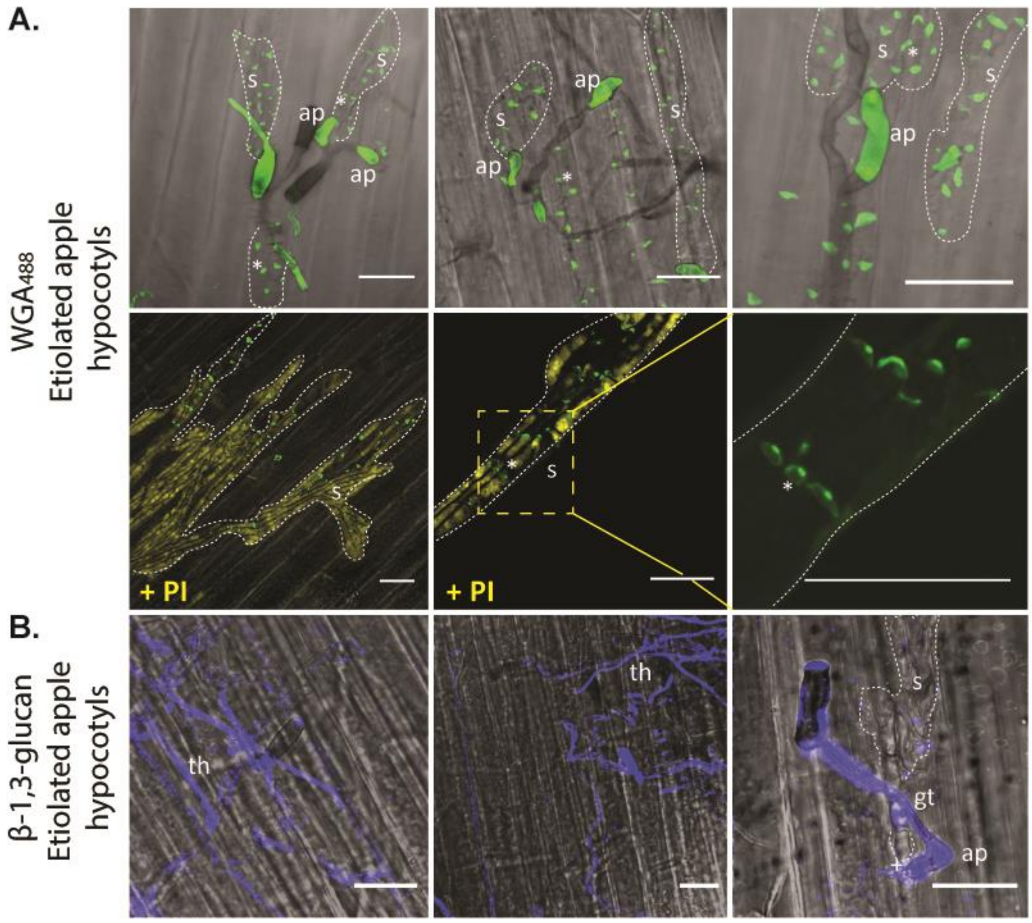


**Figure S6**. **Label-accessible chitin and β-1,3-glucan on the surface of infection structures developed by *Venturia inaequalis* in and on etiolated apple hypocotyls. A.** The fluorophore-labelled lectin $WGA_{488}$ was used to visualize chitin (green), and propidium iodide (PI, yellow) was used to stain fungal nuclei, in conjunction with confocal laser scanning microscopy. Dashed yellow squares indicate zoomed-in areas. **B.** The monoclonal anti-β-1,3-glucan primary antibody and CF-488 secondary antibody were used to label β-1,3-glucan (blue). All scale bars: 20 µm. ap, appressorium; s, stroma; th, tubular hyphae; *: septa; dashed white lines highlight stromata.

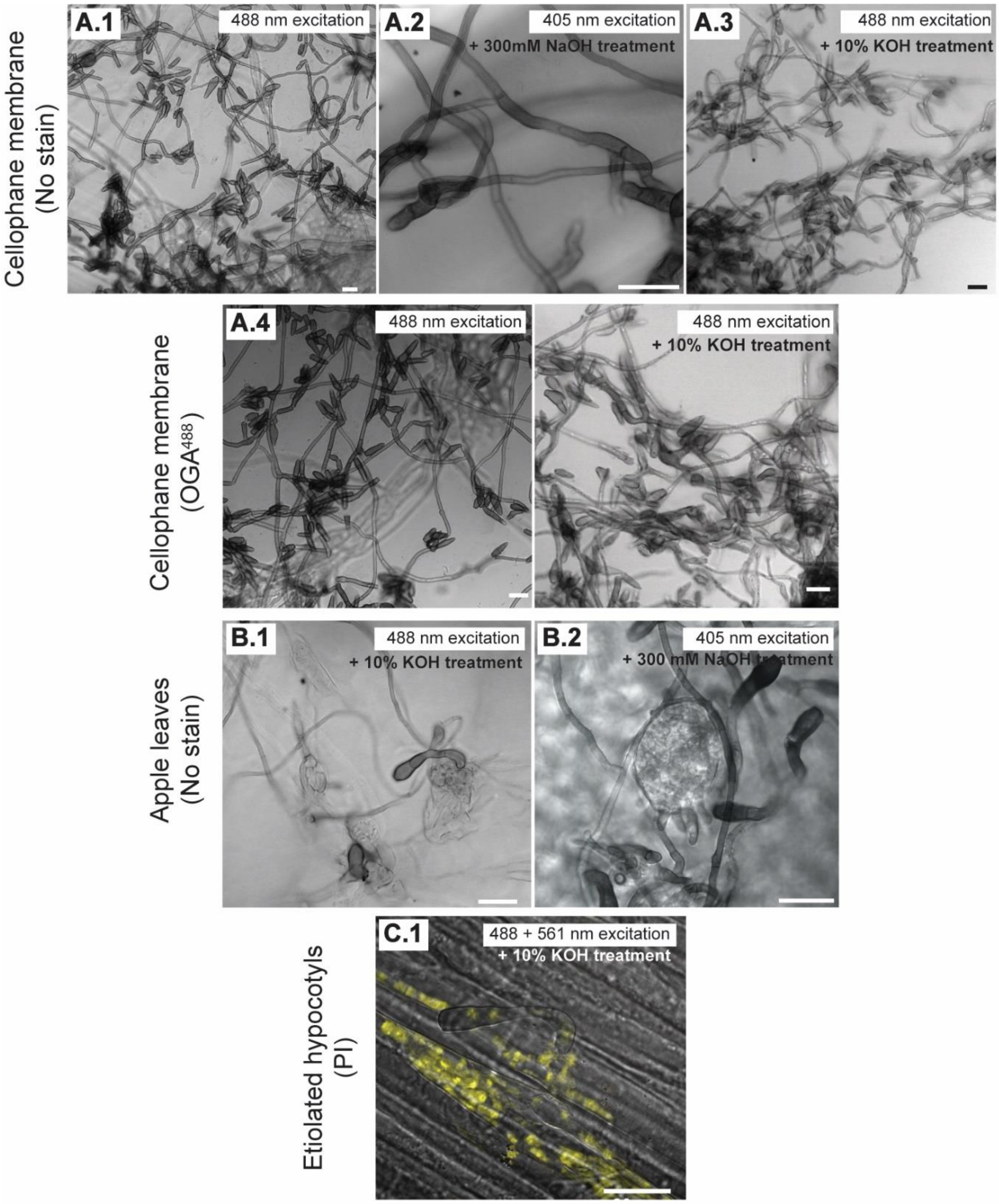


**Figure S7. Representative negative controls to ensure that the emission signals observed during confocal laser scanning microscopy were derived from specific labelling and not autofluorescence**. **A.1** Cellophane membranes (CMs) associated with *V. inaequalis* growth did not emit any signal when excited at 488 nm (autofluorescence excitation control for WGA$^{AF488}$, OGA$^{488}$, α-1,3-glucan and β-1,3-glucan labelling). **A.2** CMs associated with *V. inaequalis* growth did not emit any signal when excited at 405 nm following treatment with 300 mM NaOH (autofluorescence excitation control for calcofluor white labelling). **A.3** CMs associated with *V. inaequalis* growth treated with 10% KOH did not emit any signal when excited at 488 nm (autofluorescence excitation control for the tissue maceration treatment used *in planta*). **A.4** Surface hyphae of *V. inaequalis* did

not show any fluorescence when excited at 488 nm after labelling with $OGA^{488}$, irrespective of whether they were treated with 10% KOH (control for $OGA^{488}$ labelling of chitosan). **B.1** *V. inaequalis*-infected apple leaf tissue treated with 10% KOH did not emit any signal when excited at 488 nm (autofluorescence excitation control for the tissue maceration treatment). **B.2** *V. inaequalis*-infected apple leaf tissue treated with 300 mM NaOH did not emit any signal when excited at 405 nm (autofluorescence excitation control for calcofluor white labelling). **C.1** Following 10% KOH treatment, *V. inaequalis*-infected etiolated apple hypocotyls stained with propidium iodide (PI) only gave a PI-specific signal when excited at 561 nm. No signal was emitted following excitation at 488 nm (control for PI staining of fungal nuclei). All scale bars: 20 µm.
